# Solid State and Solution Structures of Lanthanide Nitrate Complexes of Tris-(1-napthylphosphine oxide)

**DOI:** 10.3390/molecules29112580

**Published:** 2024-05-30

**Authors:** Simon J. Coles, Laura J. McCormick McPherson, Andrew W. G. Platt, Kuldip Singh

**Affiliations:** 1 UK National Crystallography Service, Chemistry, University of Southampton, Highfield Campus, Southampton SO17 1BJ, UK; s.j.coles@soton.ac.uk (S.J.C.); l.j.mccormick@soton.ac.uk (L.J.M.M.); 2 School of Health, Education, Policing and Sciences, Staffordshire University, Science Centre, Stoke-on-Trent ST4 2DF, UK; 3 Chemistry Department, The University of Leicester, Leicester LE1 7RH, UK; ks42@le.ac.uk

**Keywords:** lanthanide, coordination complexes, tris-1-naphthylphosphine oxide, crystal structures, NMR spectroscopy

## Abstract

Coordination complexes of lanthanide metals with tris-1-naphthylphosphine oxide (Nap_3_PO, L) have not been previously reported in the literature. We describe here the formation of lanthanide(III) nitrate complexes Ln(NO_3_)_3_L_4_ (Ln = Eu to Lu) and the structures of [Ln(NO_3_)_3_L_2_]·2L (Ln = Eu, Dy, Ho, Er) and L. The core structure of the complexes is an eight-coordinate [Ln(NO_3_)_3_L_2_] with the third and fourth ligands H-bonded via their oxygen atoms to one of the naphthyl rings. The structures are compared with those of the analogous complexes of triphenylphosphine oxide and show that the Ln-O(P) bond in the Nap_3_PO complexes is slightly longer than expected on the basis of differences in coordination numbers. The reaction solutions, investigated by ^31^P and ^13^C NMR spectroscopy in CD_3_CN, show that coordination of L occurs across the lanthanide series, even though complexes can only be isolated from Eu onwards. Analysis of the ^31^P NMR paramagnetic shifts shows that there is a break in the solution structures with a difference between the lighter lanthanides (La–Eu) and heavier metals (Tb–Lu) which implies a minor difference in structures. The isolated complexes are very poorly soluble, but in CDCl_3_, NMR measurements show dissociation into [Ln(NO_3_)_3_L_2_] and 2L occurs.

## 1. Introduction

Complexes of lanthanide nitrates and halides with triphenylphosphine oxide have been studied since the 1960s [1,2], and there is a wealth of structural data available on their nitrate complexes. These generally form neutral molecular [Ln(NO_3_)_3_(Ph_3_PO)_3_], but also [Ln(NO_3_)_3_(Ph_3_PO)_2_EtOH] and ionic [Ln(NO_3_)_2_(Ph_3_PO)_4_]^+^ [3,4,5,6,7,8,9,10,11,12,13]. The molecular complexes are invariably nine-coordinate with a pseudo *mer*-octahedral arrangement around the metal (visualising the nitrates as pseudo-monodentate ligands bonded through the nitrogen) whilst the cationic complexes have a similar *trans*-octahedral geometry. There are no reports of complexes between lanthanide nitrates and related tris-1-naphthylphosphine oxide (Nap_3_PO). Indeed, there seems to be only one report of metal complexes with this ligand, [Re(CO)_3_(Nap_3_PO)]_2_[C_6_H_2_O_4_] and [Re(CO)_3_(Nap_3_PO)]_2_[C_6_Cl_2_O_4_], which were formed serendipitously during in situ oxidation of the phosphine [14]. Naphthyl-substituted phosphines are slightly more basic than the corresponding phenyl compounds [15], and it is reasonable to assume that this acts as a proxy for the basicity of the corresponding oxides. Naphthyl substituents are obviously more sterically demanding, with the cone angle of Nap_3_P being around 200° [16], and this combination of effects, together with the lack of literature on the subject, makes a study of the complexes interesting. We report here our investigations into the formation, structures in the solid state and solution properties of complexes of tris-1-naphthylphosphine oxide (L) with lanthanide nitrates.

## 2. Results and Discussion

The ligand was prepared by oxidation of the phosphine with H_2_O_2_ in acetone. Attempts to prepare lanthanide nitrate complexes in the conventional manner by heating solutions of the nitrate with a suspension of the ligand in ethanol were not successful. After refluxing for several hours, only unreacted Nap_3_PO was recovered. Reactions carried out with a large excess of lanthanide nitrate in acetonitrile in a sealed vessel at 90 °C gave, on slow cooling, yellow crystalline materials for the heavier lanthanides (from Eu onwards). The infrared spectra show typical bands from coordinated nitrate ions [17], and the P=O stretches show the expected decrease in frequency on coordination to the metal. The positions of the main bands are given in Table S1 in the Supplementary Information.

It was not possible to prepare complexes of the lighter metals La to Nd by the same method, and in the case of La(NO_3_)_3_, this method led only to the isolation of crystals of Nap_3_PO (characterised by infrared spectroscopy, elemental analysis and single-crystal X-ray diffraction). The complexes with heavier lanthanides gave elemental analysis for the bulk material corresponding to the composition Ln(NO_3_)_3_L_4_. The high ligand-to-metal ratio in the isolated complexes is surprising since the reactions were all carried out with a large excess of lanthanide nitrates. This presumably reflects a strong preference for the formation of the 1:4 complexes and/or their lower solubility.

### 2.1. Solution Studies

Solution studies by NMR spectroscopy were carried out with an excess of lanthanide nitrate (to ensure complete coordination of the ligand) with Nap_3_PO in CD_3_CN. The ratio of Ln(NO_3_)_3_·6H_2_O to Nap_3_PO ranged from about 1:1 to 7:1. With lower ratios of metal, incomplete dissolution of Nap_3_PO occurred. The spectra are shown in Figure S5, and details of the quantities of reagents and solvent in Table S3 in the Supplementary Information. In some instances, different ratios of metal to ligand were used. These made no significant difference to the spectra obtained. The results show that even though complexes could not be isolated for the lighter metals, they do form in solution. Clear evidence for this is seen in the characteristic paramagnetic shifts, particularly in the ^31^P NMR spectra, together with their strong temperature dependence, which were observed in all cases. Similarly, the ^13^C NMR spectra show shifts in the position of signals relative to the uncoordinated ligand. The NMR data are given in Table 1 and Table 2.

**Figure 1:**
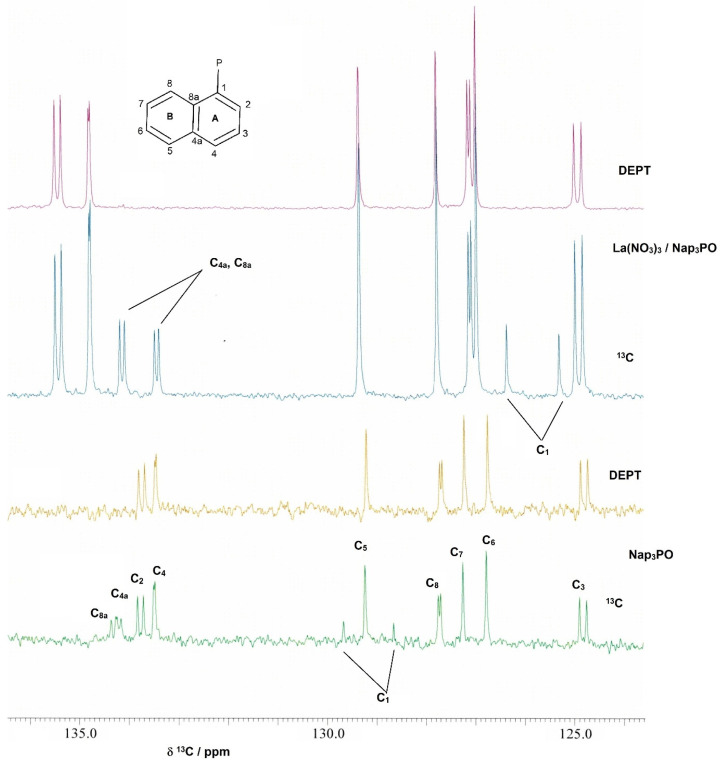
The ^13^C NMR spectra of Nap_3_PO and La(NO_3_)_3_/Nap_3_PO in CD_3_CN.

The assignments in the ^13^C NMR spectra of the complexes were made in comparison with the assignments from the ^1^H and ^13^C NMR spectra of Nap_3_PO. For Nap_3_PO, the initial assignment of H_8_ in the ^1^H spectrum was based on it being the highest frequency signal in 1-substituted naphthalenes [18]. From this, the assignment of the remaining protons and the carbons in the NMR spectra was achieved using COSY (to identify strongly coupled protons) and heteronuclear correlation spectra (to give the directly bonded carbons). The assignments were confirmed by analysis of the ^1^H–^13^C long-range correlation (HMBC) spectrum. In aromatic systems, the proton couplings to the ortho and para carbon atoms are generally small and give low intensity or completely absent cross peaks in the spectrum. Coupling to the meta carbon, however, is generally in the region of 8 Hz [19] and thus readily observed in the standard HMBC experiment. This was particularly useful in assigning C_4a_ and C_8a_. The assignments and representative spectra are given in Table S2 and Figures S1–S4, respectively, in the Supplementary Information. The main effect on coordination is on the atoms in the A ring which are closest to the coordination sites and/or paramagnetic centres. The ^13^C NMR spectra from the La(NO_3_)_3_/Nap_3_PO system in CD_3_CN, from which only unreacted Nap_3_PO could be recovered, are shown in Figure 1. Evidence of coordination is seen here particularly in the shift of C_1_ (clearly observed as a doublet with no attached hydrogen) which shifts over 3 ppm to a higher field on coordination.

The isolated complexes are very poorly soluble in common organic solvents. Thus, attempts to obtain spectra of the isolated [Ln(NO_3_)_3_L_2_] 2L in CD_3_CN in the absence and presence of added Ln(NO_3_)_3_ (to bind to the non-coordinated ligands) were not successful. The ^31^P NMR spectra can be obtained in CDCl_3_ in some instances, on prolonged accumulation at 30 °C and 60 °C, and show two broad signals in an approximately 1:1 ratio, one due to free ligand at about 41 ppm and the other being a broader signal for the lanthanide-coordinated molecule. This implies that the complexes dissolve as [Ln(NO_3_)_3_L_2_] + 2L. The chemical shifts of the coordinated ligands are very different between CD_3_CN and CDCl_3_, which implies that different lanthanide-containing species are being observed. It might be anticipated that the core [Ln(NO_3_)_3_L_2_] moiety is present in CD_3_CN solution due to the excess of lanthanide ions present, but the very different chemical shifts would imply that this is not the case. The chemical shifts in CD_3_CN are also very different from the average of the two signals observed in CDCl_3_. It is thus unlikely that rapid exchange between [Ln(NO_3_)_3_L_2_] and L is being observed in the spectra in CD_3_CN. It is possible that other exchange processes occur; for example, ionisation of one of the nitrates to give [Ln(NO_3_)_2_L_2_(CD_3_CN)_n_]^+^ in equilibrium with an unionised [Ln(NO_3_)_3_L_2_] complex may be occurring in the more polar solvent. Alternatively, species based on the coordination of a single Nap_3_PO as a result of an excess of Ln(NO_3_)_3_ may be present. There is, however, no direct evidence for any of these possibilities, and thus, explanations remain tentative.

**Figure 2:**
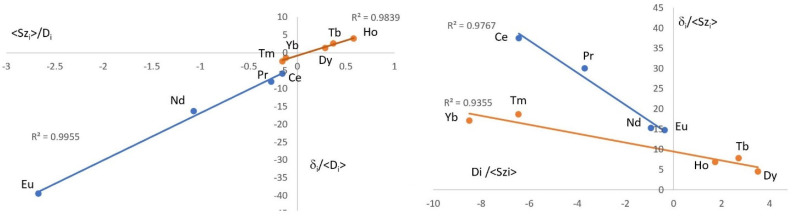
Lanthanide-induced ^31^P NMR shift plots for complexes of Nap_3_PO in CD_3_CN at 30 °C.

Analysis of lanthanide-induced shifts is an established method used to deduce whether solution structures remain constant across the lanthanide series. Analyses based on the observation of one, two and three nuclei in a complex have been developed [20,21]. Here, due to a combination of low solubility and excessive line broadening, particularly for the heavier lanthanides, only the phosphorus nucleus was observable for all the complexes studied, and hence the single-nucleus analysis of lanthanide-induced shifts was performed. In this method, plots of δ_i_/D_i_ vs. <S_z_>_i_/D_i_ and δ_i_/<S_z_>_i_ vs. D_i_/<S_z_>_i_ are analysed, where δ_i_ is the paramagnetic shift for a given complex where δ_i_ = δ_Ln_ − ½[δ_La_ − δ_Lu_] and δ_Ln,_ δ_La_ and δ_Lu_ are the observed shifts for the lanthanide, lanthanum and lutetium complexes, respectively. <S_z_>_i_ is the spin expectation value for a particular lanthanide ion, and D_i_ is the Bleaney factor for the lanthanide ion which depends only on its electronic configuration. Both plots are expected to be linear if there is structural uniformity in solution across the series, whilst breaks in one or both imply minor or major structural changes, respectively [22]. For complexes of Nap_3_PO, analysis has been carried out on the data at 30 °C and at 80 °C in CD_3_CN and indicates that at both temperatures there is a change in structure at the middle of the series. Thus, plots of δ_i_/D_i_ vs. <S_z_>_i_/D_i_ and δ_i_/<S_z_>_i_ vs. Di/<S_z_>_i_ both show separate good linear trends for Ce–Eu and Tb–Yb as shown in Figure 2. The plots of δ_i_/<S_z_>_i_ vs. Di/<S_z_>_I_ show a clear break. The plots of δ_i_/D_i_ vs. <S_z_>_i_/D_i_ could also be considered to have a break at the same point. However, it must be pointed out that a best-fit line through all points also gives an acceptable fit (R^2^ = 0.9894), and it is probable that the change in solution structure is thus a minor one. It is interesting to note that the break in the plots coincides with the region where solid complexes of the lighter metals could not be isolated.

### 2.2. Crystal Structures of Nap_3_PO and Its Complexes

**Figure 3:**
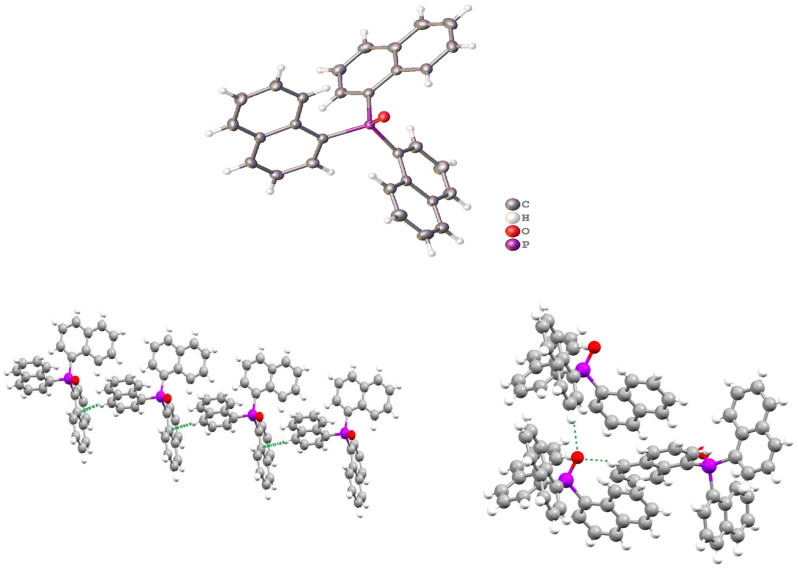
The structure of Nap_3_PO (**upper**), thermal ellipsoids drawn at 50%, one of the edge-to-face intermolecular interactions (**lower left**) in Nap_3_PO and the hydrogen bonding of the PO group (**lower right**). The interactions are indicated by dashed lines other weak intermolecular C⋯·H interactions are illustrated in Figure S5 in the Supplementary Information.

The crystal structure of Nap_3_PO.mesitylene has been previously determined [14] and is very similar to that obtained here from the recovery of unreacted Nap_3_PO in the reaction with lanthanum nitrate in acetonitrile. Its structure is shown in Figure 3. The P=O distance is marginally shorter in the mesitylene adduct, 1.451 Å compared to 1.493 Å, whilst the other distances and angles around the phosphorus atom are essentially the same. There is hydrogen bonding between the P=O group and H3 and H5 on two adjacent molecules (see Figure 3). The O⋯⋯·H distances are ~2.290 Å (to H3) and ~2.602 Å (to H5) compared with the sum of van der Waals radii of 3.05 Å and thus represent a relatively strong hydrogen bond. The packing can be described in terms of a collection of weak face-to-face and edge-to-face C-H aromatic interactions. The example shown in Figure 3 is of an edge-to-face interaction where the H4⋯·C8a distances are ~2.88 Å (sum of the C/H van der Waals radii is 2.98 Å).

Solid complexes suitable for X-ray crystallography were obtained for a range of metals (Ln = Eu, Dy, Ho, Er). Crystals of the Yb and Lu complexes were of poor quality and hence did not provide satisfactory crystal data. The structures show that the complexes crystallise as [Ln(NO_3_)_3_(Nap_3_PO)_2_]·2Nap_3_PO where the metal is eight-coordinate with two Nap_3_PO ligands on opposite sides and nitrates occupying an “equatorial” belt. The remaining two ligands are not coordinated to the lanthanide ion. Full details of the data collection and refinement together with complete listings of bond distances and angles are given in Tables S4–S11 and associated text in the Supplementary Information.

**Figure 4:**
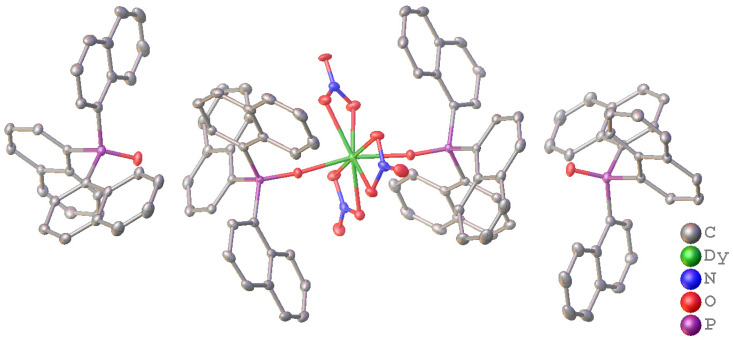
The structure of [Dy(NO_3_)_3_(Nap_3_PO)_2_]·2Nap_3_PO. Thermal ellipsoids drawn at 50%, hydrogen atoms omitted for clarity.

The geometry around the metal was determined by continuous shape measures [23,24]. This sums the deviations (S) of the atoms from their positions in a set of idealised polyhedra. Values of S below 3 represent small distortions from the standard polyhedron. The best descriptions for the Eu, Dy and Ho complexes are distorted hexagonal bipyramids (Eu (S = 4.51), Dy (S = 4.74) and Ho (S = 5.02)), and the best description for Er is cubic (S = 3.76). The structure of the Dy complex is shown as a representative example in Figure 4.

**Figure 5:**
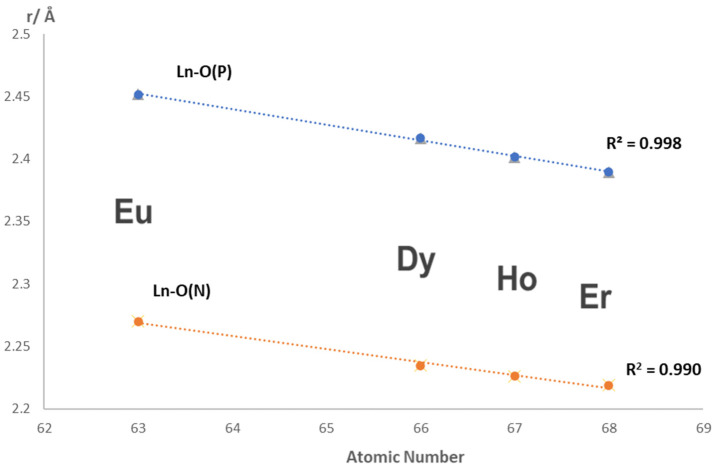
Ln-O bond distance as a function of atomic number in [Ln(NO_3_)_3_(Nap_3_PO)_2_]·2Nap_3_PO.

Both the (P)O-Ln and (N)O-Ln bond distances decrease in a linear manner (R^2^ ≥ 0.99) with atomic number from Eu to Er as expected based on the lanthanide contraction as shown in Figure 5.

There are obvious differences between complexes of Ph_3_PO and Nap_3_PO which are primarily due to the increased steric requirements. This prohibits the formation of the pseudo *cis* arrangement common in Ph_3_PO complexes and allows only two Nap_3_PO to coordinate to the lanthanide ions, resulting in a coordination number of 8.

**Figure 6:**
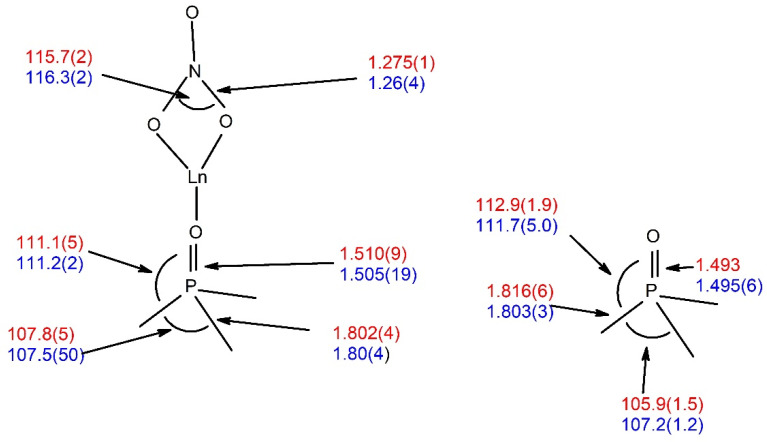
Average bond lengths (Å) and angles (°) in Nap_3_PO (red), Ph_3_PO (blue) and their lanthanide nitrate complexes.

On coordination to the lanthanide ions, the expected increase in P=O distance is observed from 1.493 Å in Nap_3_PO to an average of 1.513 (1) in the complexes. This increase is slightly greater than that found for the rhenium complex (1.502 Å) [14] and probably reflects the higher charge on the lanthanide ion. There are similarly small changes to the angles within the Nap_3_PO ligand on coordination with a small narrowing of O–P–C (from 113.0° to 111.1°) and a widening of C–P–C (105.9° to 107.8°). These changes are very similar to those found in analogous triphenylphosphine oxide complexes with lanthanide nitrates which are summarised in Figure 6, and it thus appears that the increased steric effect in Nap_3_PO does not significantly alter the structure of the ligand on coordination.

There is H-bonding between the oxygen of the non-coordinated Nap_3_PO and H2 on one of the naphthyl rings. The distance is ~2.592 Å and is significantly shorter than the sum of the O/H van der Waals radii (3.05 Å) [25].

**Figure 7:**
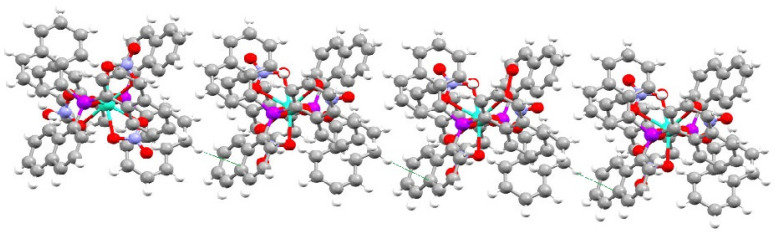
The interaction between H4 and C5 (dashed line) in [Dy(NO_3_)_3_(Nap_3_PO)_2_]·2Nap_3_PO.

There are weak intermolecular aromatic interactions similar to those found in Nap_3_PO. For instance, the H4····C5 distance shown in Figure 7 is ~2.872 Å.

The coordination geometries are similar to those found in complexes of lanthanide nitrates with the sterically demanding ^t^Bu_3_PO [26] which have subsequently been investigated as single-molecule magnets [27]. It is interesting to note in this respect that tricyclohexyl phosphine oxide (Cy_3_PO), for which the cone angle of the parent phosphine is 170°, forms 3:1 complexes [Ln(NO_3_)_3_(Cy_3_PO)_3_] [28]. The bulkier ^t^Bu_3_PO (the cone angle for ^t^Bu_3_P is 182°) and Nap_3_PO form only 2:1 complexes presumably because of steric effects. Although a direct comparison of the Ln-O bond distances in the Nap_3_PO complexes with those in analogous complexes of Ph_3_PO is not possible because of the differing ionic radii and coordination numbers, correction for these variables is relatively straightforward. The results of this analysis are given in Table 3.

Subtraction of the ionic radii for the nine- and eight-coordinate radii from the observed Ln-O distances would be expected to give the same value. This is clearly seen along each row in Table 3. The values for the (N)O-Ln distances are the same for both Ph_3_PO and Nap_3_PO complexes, indicating that the bonding to the nitrate is largely unaffected by the presence of the bulkier Nap_3_PO. The (P)O-Ln distances are significantly different, with the bond lengths to Nap_3_PO being slightly longer than for the Ph_3_PO complexes. This most likely arises as a result of the increased steric demands of the Nap_3_PO compared to Ph_3_PO.

## 3. Materials and Methods

### 3.1. Crystallography

Suitable crystals of each compound were selected, coated in protective perfluoroether oil and mounted on a MiTeGen holder. Diffraction data were collected on a Rigaku FRE+ diffractometer equipped with VHF Varimax confocal mirrors and an AFC12 goniometer and HyPix 6000HE detector or a Rigaku 007HF equipped with Varimax confocal mirrors and an AFC11 goniometer and HyPix 6000 detector. The crystals were kept at a steady *T* = 100(2) K during data collection. The structure was solved with the **ShelXT** [29,30] structure solution program using the Intrinsic Phasing solution method and by using **Olex2** [30] as the graphical interface. The model was refined with version 2014/7 of **ShelXL** [31] using least squares minimisation. The total number of runs and images was based on the strategy calculation from the program **CrysAlisPro** [32]. All non-hydrogen atoms were refined with anisotropic thermal displacement parameters. Aromatic and aliphatic hydrogen atoms have been included at their geometrically estimated positions. Model refinement was unremarkable. Further details may, in all cases, be found in the cifs. Further details about the modelling of the disorder may be found in the Supporting Information.

Crystallographic data for the structures in this paper have been deposited with the Cambridge Crystallographic Data Centre as supplementary publication numbers CCDC 2334501-2334505 for the Dy, Er, Eu, Ho complexes and Nap_3_PO, respectively. Copies of the data can be obtained, free of charge, on application to CCDC, 12 Union Road, Cambridge CB2 1EZ, UK (Fax: +44(0)-1223-336033 or e-mail: deposit@ccdc.cam.ac.uk).

### 3.2. NMR Spectroscopy

NMR spectra were recorded at 30 °C in CDCl_3_ or CD_3_CN solution on a JEOL ECX 400 using approximately 1 mg of solid in 1 mL of the appropriate deuterated solvent. The ^1^H NMR spectra were measured at 400 MHz, whilst the ^31^P and ^13^C spectra were recorded at 161.9 MHz and 100.5 MHz, respectively. For examination of the reaction solutions, about 4–7 mg of Nap_3_PO and 10–20 mg Ln(NO_3_)_3_·6H_2_O were dissolved in about 0.5 g CD_3_CN. For some samples, extended accumulations of the order of one day were required due to extremely large linewidths.

### 3.3. Infrared Spectroscopy

Infrared spectra were recorded with a resolution of ±2 cm^−1^ on a Thermo Nicolet Avatar 370 FT-IR spectrometer operating in ATR mode. The samples were compressed onto the optical window, and spectra were recorded without further sample pre-treatment.

### 3.4. Synthesis

**Nap_3_PO** Nap_3_P (9.86 g, 23 mmol) was stirred in 100 mL acetone cooled in an ice bath, and H_2_O_2_ (3.50 g 30% solution, 31 mmol) in 25 mL acetone was added over 10 min. The mixture was stirred at 0 °C for 2 h and allowed to warm to room temperature. Stirring was continued for 1 week until the ^31^P NMR of the mixture showed a single peak at 40.0 ppm with no signal from unreacted phosphine at −34 ppm. The mixture was filtered, washed with water and ethanol, and dried at the pump to give the oxide, 9.15 g (93%) as a white powder. NMR ^31^P (161.9 MHz, CDCl_3_) 41.1, ^13^C (100.5 MHz, CDCl_3_) 124.5 (CH)(d) *J*_PC_ 14.4 Hz, 126.6 (CH)(s), 127.6 (CH)(s), 128.3 (CH)(d) *J*_PC_ 5.8 Hz, 128.9 (CH)(s), 128.9 (CP)(d) ^1^*J*_PC_ 104.4 Hz, 133.4 (CH)(d) *J*_PC_ 2.9 Hz, 133.8 (CH)(d) *J*_PC_ 12.5 Hz, 134.1 (C)(d) *J*_PC_ 8.6 Hz, 134.4 (C)(d) *J*_PC_ 7.6 Hz, NMR ^31^P (161.9 MHz, CD_3_CN) 39.1, ^13^C (100.5 MHz, CD_3_CN) 124.7 (CH)(d) *J*_PC_ 14.4 Hz, 126.7 (CH)(s), 127.2 (CH)(s), 127.6 (CH)(d) *J*_PC_ 8.6 Hz, 129.1 (CP)(d) ^1^*J*_PC_ 101.6 Hz, 129.1 (CH)(s), 133.4 (CH)(d) *J*_PC_ 2.8 Hz, 133.7 (CH)(d) *J*_PC_ 11.5 Hz, 134.1 (C)(d) *J*_PC_ 7.6 Hz, 134.2 (C)(d) *J*_PC_ 9.5 Hz.

Analysis % required (found) Nap_3_PO C 84.10 (83.44) H 4.94 (4.93) N 0.00 (0.10).

Complexes of Nap_3_PO were prepared by reacting acetonitrile solutions of excess lanthanide nitrates with Nap_3_PO in a sealed vessel. The ligand gradually dissolved on heating between 80 and 90 °C. Representative examples are given below.

Dy(NO_3_)_3_·6H_2_O (0.14 g, 0.31 mmol) was dissolved in 4 mL CH_3_CN, and Nap_3_PO (0.1033 g, 0.24 mmol) was added. The mixture was heated to between 80 and 90 °C for 5 h, after which a clear yellow solution was obtained. Slow cooling to room temperature gave yellow crystals of Dy(NO_3_)_3_(Nap_3_PO)_4_ 0.0907 g (78% based on Nap_3_PO).

Analysis % required (found) Dy(NO_3_)_3_(Nap_3_PO)_4_ C 69.89 (69.26) H 4.11 (4.10) N 2.04 (2.01).

Er(NO_3_)_3_·6H_2_O (0.16 g, 0.34 mmol) was dissolved in 4 mL CH_3_CN, and Nap_3_PO (0.06 g, 0.15 mmol) was added. The mixture was heated to between 80 and 90 °C for 16 h, after which a clear yellow solution was obtained. Slow cooling to room temperature gave yellow crystals of Er(NO_3_)_3_(Nap_3_PO)_4_ 0.05 g (67% based on Nap_3_PO).

Analysis % required (found) Er(NO_3_)_3_(Nap_3_PO)_4_ C 69.72 (69.48) H 4.10 (4.09) N 2.03 (2.03).

Yb(NO_3_)_3_·6H_2_O (0.10 g, 0.23 mmol) was dissolved in 5 mL CH_3_CN, and Nap_3_PO (0.30 g, 0.71 mmol) was added. The mixture was heated to between 80 and 90 °C for 5 h, after which a clear yellow solution was obtained. Slow cooling to room temperature gave yellow crystals of Yb(NO_3_)_3_(Nap_3_PO)_4_ 0.31 g (83% based on Nap_3_PO).

Analysis % required (found) Yb(NO_3_)_3_(Nap_2_MePO)_4_ C 69.53 (69.46) H 4.08 (4.05) N 2.03 (1.98).

Lu(NO_3_)_3_·6H_2_O (0.141 g, 0.3 mmol) was dissolved in 3 mL CH_3_CN, and Nap_3_PO (0.045 g, 0.1 mmol) was added. The mixture was heated to between 80 and 90 °C for 5 h, after which a clear yellow solution was obtained. Slow cooling to room temperature gave yellow crystals of Ln(NO_3_)_3_(Nap_3_PO)_2_·Nap_3_PO 0.0392 g (70% based on Nap_3_PO).

Analysis % required (found) Lu(NO_3_)_3_(Nap_3_PO)_4_ C 69.47 (68.93) H 4.08 (4.03) N 2.03 (2.12).

## 4. Conclusions

The formation of complexes between Nap_3_PO and lanthanide nitrates in CD_3_CN for all Ln studied has been established by NMR spectroscopy (^13^C and ^31^P), although their nature in solution is uncertain. Coordination complexes can be isolated for Ln = Eu to Lu and have the composition Ln(NO_3_)_3_L_4_. Single-crystal X-ray crystallography shows that these 4:1 complexes have structures based on [Ln(NO_3_)_3_ (Nap_3_PO)_2_]·2L in all cases with two Nap_3_PO not directly coordinated to the metal, although they are hydrogen bonded to the naphthyl ring. The complexes differ from the less sterically demanding Ph_3_PO complexes in that only two phosphine oxides are directly attached to the lanthanide ion. Although the isolated complexes are poorly soluble, in some cases, ^31^P NMR spectroscopy in CDCl_3_ has established that they dissolve as [Ln(NO_3_)_3_ (Nap_3_PO)_2_] + 2Nap_3_PO.

## Figures and Tables

**Table 1:** ^31^P NMR data in CD_3_CN and CDCl_3_.

	CD_3_CN ^a^				CDCl_3_ ^b^							
	30 °C		80 °C		30 °C				60 °C			
	δ ^c^	w_1/2_ ^d^	δ ^c^	w_1/2_ ^d^	δ_free_ ^c^	w_1/2_ ^d^	δ_coord_ ^c^	w_1/2_ ^d^	δ_free_ ^c^	w_1/2_ ^d^	δ_coord_ ^c^	w_1/2_ ^d^
La	42.3	19	42.9	13								
Ce	79.3	65	73.9	44								
Pr	119.7	200	113.2	120								
Nd	111.1	300	108.9	100								
Eu	−115.2	450	−99.2	400	41.8	80	Not Obs		40.3	45	−67.6	300
Tb	−206	5500	−185	1100								
Dy	−86.6	2500	−94	600	41.0	40	−11.2	4500	40.4	110	−52.1	1300
Ho	−113.3	3000	−111.1	750	41.0	175	−204.0	600				
Er					41.0	60	−235.7	200	40.3	40.3	−198.5	
Tm	−110.2	2000	−85.8	300								
Yb	−1.6	1600	13.1	1000	40.8	75	31.8	440	40.1	150	33.7	1000
Lu	42.8	20	43.8	13	40.8	30	49.2	190	40.3	180	47.9	200
Nap_3_PO ^b^	39.2	7			41.1	12			40.1	7		

^a^ Reaction solution with excess lanthanide nitrate; ^b^ isolated complex [Ln(NO_3_)_3_L_2_]·2L; ^c^ δ/ppm; ^d^ w_1/2_ line width at half height/Hz.

**Table 2:** ^13^C NMR chemical shifts and coupling constants for Nap_3_PO and selected complexes in CD_3_CN at 30 °C.

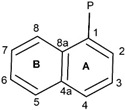										
δ (ppm)/*J*_C–P_ (Hz)										
	C_1_	C_2_	C_3_	C_4_	C_4a_	C_5_	C_6_	C_7_	C_8_	C_8a_
Nap_3_PO	129.0/101.6	133.7/11.5	124.7/14.4	133.4/2.8	134.1/7.6	129.1	126.7	127.2	127.6/5.8	134.2/9.5
La	125.8/106.4	135.3/12.5	124.8/15.3	134.7/2.9	133.4/7.7	129.2	126.9	127.7	127.0/5.7	134.1/9.5
Ce	127.6/104.4	136.5/12.5	125.3/14.4	135.0/2.9	134.3/8.6	129.2	126.7	127.3	127.1/2.4	134.4/10.4
Pr	130.7/111.2	139.1/12.5	126.3/15.4	135.3	135.1	129.5	126.7	127.1	128.3/4.8	136.5
Nd	123.9/126.5	135.0/14.4	125.1/11.5	136.1	133.6	129.3	127.4	126.8	126.2	134.0
Eu	128.6/110.3	133.9/12.5	124.5/15.3	134.4	132.1/7.6	129.2	127.1	127.9	127.8	133.6/8.6
Lu	125.8/107.3	135.3/12.5	124.9/14.8	136.7/2.9	133.2/8.6	129.3	126.9	127.6	127.0/5.8	133.9/9.5

Spectra recorded at 80 °C in CD_3_CN from solutions of L plus excess Ln(NO_3_)_3_.

**Table 3:** Comparison of the bond distances (Å) in Ph_3_PO and Nap_3_PO complexes of lanthanide nitrates corrected for ionic radii of the lanthanide.

		Eu	Dy	Ho	Er	Average
PO-Ln-r ^a^	Ph_3_PO	1.199	1.199	1.185	1.194	1.194(6)
PO-Ln-r ^b^	Nap_3_PO	1.204	1.208	1.213	1.212	1.209(4)
NO-Ln-r ^b^	Ph_3_PO	1.378	1.389	1.377	1.389	1.383(5)
NO-Ln-r ^b^	Nap_3_PO	1.386	1.390	1.387	1.386	1.387(2)

^a^ Nine-coordinate ionic radii; ^b^ eight-coordinate ionic radii.

## Data Availability

All data is available on request to the corresponding author.

## Supplementary Materials

The following supporting information can be downloaded at: https://www.mdpi.com/article/10.3390/molecules29112580/s1, Figure S1: The ^13^C (left) and ^1^H (right) NMR spectra of Nap_3_PO in CDCl_3_ at 30 °C; Figure S2: The COSY (left) and the ^1^H–^13^C HETCOR (right) spectra of Nap_3_PO in CDCl_3_ at 30 °C; Figure S3: The HMBC spectrum of Nap_3_PO in CDCl_3_ at 30 °C; Figure S4: The ^13^C spectra of the reaction solutions in CD_3_CN; Figure S5: The ^31^P NMR spectra of the reaction solutions of excess Ln(NO_3_)_3_ with Nap_3_PO in CD_3_CN at 30 °C: Figure S6: Packing diagrams for Nap_3_PO; Table S1: Infrared Positions /cm^−1^ of Nitrate and PO bands in Nap_3_PO complexes; Table S2: ^1^H and ^13^C NMR data for Nap_3_PO in CDCl_3_ and CD_3_CN at 30 °C; Table S3: Mass and volumes of reagents used in the NMR experiments; Table S4: Bond Lengths in Å for Eu(NO_3_)_3_(Nap_3_PO)_3_; Table S5: Bond Angles in Å for Eu(NO_3_)_3_(Nap_3_PO)_3_; Table S6: Bond Lengths in Å for Dy(NO_3_)_3_(Nap_3_PO)_3_; Table S7: Bond Angles in Å for Dy(NO_3_)_3_(Nap_3_PO)_3_; Table S8: Bond Lengths in Å for 2023NCS0640_2a. Table S9: Bond Angles in Å for Ho(NO_3_)_3_(Nap_3_PO)_3_; Table S10: Bond Lengths in Å for Er(NO_3_)_3_(Nap_3_PO)_3_; Table S11: Bond Angles in Å for Er(NO_3_)_3_(Nap_3_PO)_3_.
