# Supplementary material for: Solid State and Solution Structures of Lanthanide Nitrate Complexes of Tris-(1-napthylphosphine oxide)
Source: Molecules. 2024 May 30;29(11):2580. doi: 10.3390/molecules29112580 (PMC11173986; doi:10.3390/molecules29112580)

checkCIF/PLATON report

Structure factors have been supplied for datablock(s) 2023ncs0641_1a

THIS REPORT IS FOR GUIDANCE ONLY. IF USED AS PART OF A REVIEW PROCEDURE FOR PUBLICATION, IT SHOULD NOT REPLACE THE EXPERTISE OF AN EXPERIENCED CRYSTALLOGRAPHIC REFEREE.

No syntax errors found. CIF dictionary Interpreting this report

Datablock: 2023ncs0641_1a

Bond precision: C-C = 0.0020 A Wavelength=0.71073

Cell:	a=12.0971(1)	b=13.3227(1)	c=16.3187(1)
	alpha=94.401(1)	beta=94.449(1)	gamma=115.136(1)
Temperature:	100 K		

	Calculated	Reported
Volume	2356.19(4)	2356.19(4)
Space group	P -1	P -1
Hall group	-P 1	-P 1
Moiety formula	C60 H42 Eu N3 O11 P2, 2(C30 H21 O P)	C60 H42 Eu N3 O11 P2, 2(C30 H21 O P)
Sum formula	C120 H84 Eu N3 O13 P4	C120 H84 Eu N3 O13 P4
Mr	2051.75	2051.74
Dx,g cm-3	1.446	1.446
Z	1	1
Mu (mm-1)	0.804	0.804
F000	1052.0	1052.0
F000'	1052.66	
h,k,lmax	17,19,23	17,19,23
Nref	14379	14378
Tmin,Tmax	0.899,0.938	0.901,0.955
Tmin'	0.858	

Correction method= # Reported T Limits: Tmin=0.901 Tmax=0.955
AbsCorr = ANALYTICAL

Data completeness= 1.000 Theta(max)= 30.508

R(reflections)= 0.0279(12777) wR2(reflections)= 0.0708(14378)

S = 1.051 Npar= 694

The following ALERTS were generated. Each ALERT has the format
test-name_ALERT_alert-type_alert-level.
Click on the hyperlinks for more details of the test.

Alert level C

```
PLAT601_ALERT_2_C Unit Cell Contains Solvent Accessible VOIDS of .         34 Ang**3
```

Alert level G

```
PLAT154_ALERT_1_G The s.u.'s on the Cell Angles are Equal ..(Note)      0.001 Degree
PLAT300_ALERT_4_G Atom Site Occupancy of Eu1        Constrained at        0.5 Check
PLAT300_ALERT_4_G Atom Site Occupancy of O11        Constrained at        0.5 Check
PLAT300_ALERT_4_G Atom Site Occupancy of O12        Constrained at        0.5 Check
PLAT300_ALERT_4_G Atom Site Occupancy of O13        Constrained at        0.5 Check
PLAT300_ALERT_4_G Atom Site Occupancy of O21        Constrained at        0.5 Check
PLAT300_ALERT_4_G Atom Site Occupancy of O22        Constrained at        0.5 Check
PLAT300_ALERT_4_G Atom Site Occupancy of O23        Constrained at        0.5 Check
PLAT300_ALERT_4_G Atom Site Occupancy of O31        Constrained at        0.5 Check
PLAT300_ALERT_4_G Atom Site Occupancy of O32        Constrained at        0.5 Check
PLAT300_ALERT_4_G Atom Site Occupancy of O33        Constrained at        0.5 Check
PLAT300_ALERT_4_G Atom Site Occupancy of N10        Constrained at        0.5 Check
PLAT300_ALERT_4_G Atom Site Occupancy of N20        Constrained at        0.5 Check
PLAT300_ALERT_4_G Atom Site Occupancy of N30        Constrained at        0.5 Check
PLAT301_ALERT_3_G Main Residue  Disorder .............(Resd  1  )         17% Note
PLAT432_ALERT_2_G Short Inter X...Y Contact  O13      ..C44      .       2.92 Ang.
                                            2-x,1-y,1-z  =      2_766 Check
PLAT432_ALERT_2_G Short Inter X...Y Contact  C13      ..C13      .       3.09 Ang.
                                            2-x,2-y,1-z  =      2_776 Check
PLAT789_ALERT_4_G Atoms with Negative _atom_site_disorder_group  #         13 Check
PLAT790_ALERT_4_G Centre of Gravity not Within Unit Cell: Resd.  #          2 Note
              C30 H21 O P
PLAT822_ALERT_4_G CIF-embedded .res Contains Negative PART Numbers          2 Check
PLAT910_ALERT_3_G Missing # of FCF Reflection(s) Below Theta(Min).          1 Note
PLAT978_ALERT_2_G Number C-C Bonds with Positive Residual Density.         21 Info
```

0 **ALERT level A** = Most likely a serious problem - resolve or explain
0 **ALERT level B** = A potentially serious problem, consider carefully
1 **ALERT level C** = Check. Ensure it is not caused by an omission or oversight
22 **ALERT level G** = General information/check it is not something unexpected

1 ALERT type 1 CIF construction/syntax error, inconsistent or missing data
4 ALERT type 2 Indicator that the structure model may be wrong or deficient
2 ALERT type 3 Indicator that the structure quality may be low
16 ALERT type 4 Improvement, methodology, query or suggestion
0 ALERT type 5 Informative message, check

It is advisable to attempt to resolve as many as possible of the alerts in all categories. Often the minor alerts point to easily fixed oversights, errors and omissions in your CIF or refinement strategy, so attention to these fine details can be worthwhile. In order to resolve some of the more serious problems it may be necessary to carry out additional measurements or structure refinements. However, the purpose of your study may justify the reported deviations and the more serious of these should normally be commented upon in the discussion or experimental section of a paper or in the "special_details" fields of the CIF. checkCIF was carefully designed to identify outliers and unusual parameters, but every test has its limitations and alerts that are not important in a particular case may appear. Conversely, the absence of alerts does not guarantee there are no aspects of the results needing attention. It is up to the individual to critically assess their own results and, if necessary, seek expert advice.

Publication of your CIF in IUCr journals

A basic structural check has been run on your CIF. These basic checks will be run on all CIFs submitted for publication in IUCr journals (*Acta Crystallographica*, *Journal of Applied Crystallography*, *Journal of Synchrotron Radiation*); however, if you intend to submit to *Acta Crystallographica Section C* or *E* or *IUCrData*, you should make sure that full publication checks are run on the final version of your CIF prior to submission.

Publication of your CIF in other journals

Please refer to the *Notes for Authors* of the relevant journal for any special instructions relating to CIF submission.

PLATON version of 06/07/2023; check.def file version of 30/06/2023

Datablock 2023ncs0641_1a - ellipsoid plot

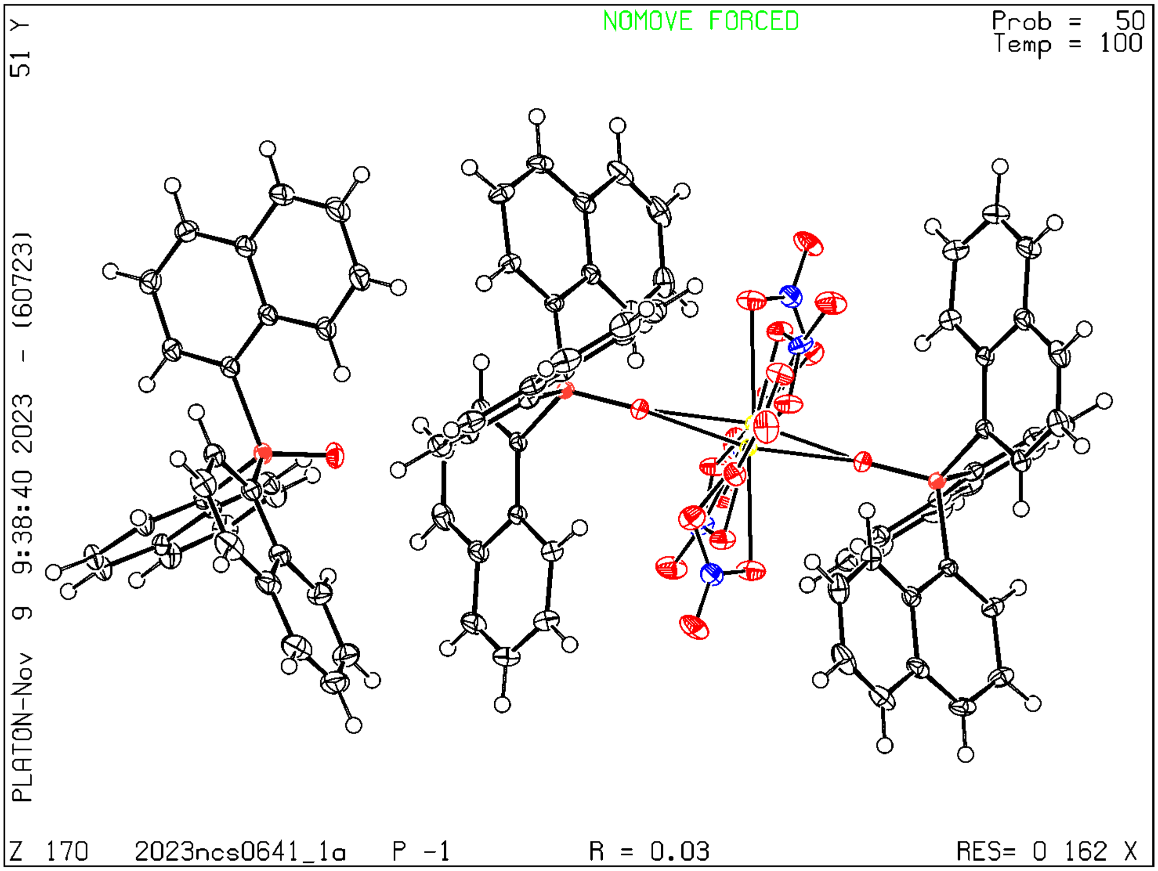

Supplement: Supplementary file 1 [file molecules-29-02580-s001.zip › molecules-3017195-supplementary/Revised Supplementary information and CIFS and cif checks/Eu cif check.pdf]
