# Supplementary material for: Solid State and Solution Structures of Lanthanide Nitrate Complexes of Tris-(1-napthylphosphine oxide)
Source: Molecules. 2024 May 30;29(11):2580. doi: 10.3390/molecules29112580 (PMC11173986; doi:10.3390/molecules29112580)
Supplement: Supplementary file 1 [file molecules-29-02580-s001.zip › molecules-3017195-supplementary/Revised Supplementary information and CIFS and cif checks/Supplementary Information.pdf]

Supplementary Information

1. **Infrared spectra**
2. **NMR spectroscopy**
3. **Crystallography**

1. Infrared Data

Table S1 Infrared Positions /cm^{-1} of Nitrate and PO bands in Nap_3PO complexes

	Eu	Tb	Dy	Ho	Er	Yb	Lu	Nap_3PO
ν_5	1503	1504	1504	1504	1508	1504	1503	
ν_1	1280<br>1267	1281<br>1267	1282<br>1267	1284<br>1267	1284<br>1267	1285<br>1267	1288<br>1266	
ν_2	1023	1024	1024	1020	1020	1020	1020	
ν_{PO}	1092	1094	1096	1098	1100	1102	1103	1141

NMR Spectroscopy

The ^{1}H and ^{13}C NMR spectra and the atom numbering of Nap_3PO are shown in figure A

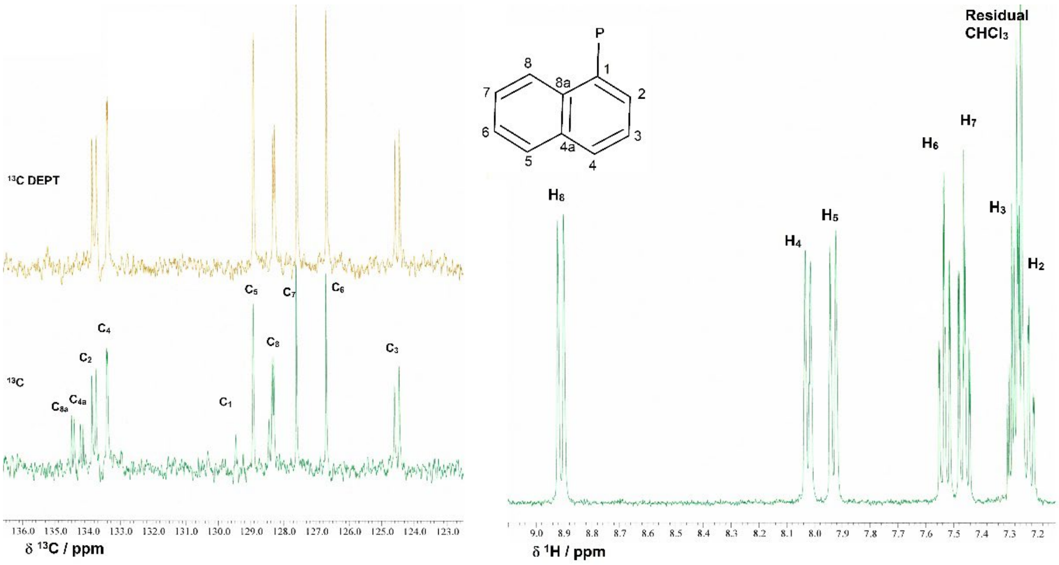

Figure S1 The ^{13}C (left) and ^{1}H (right) NMR spectra of Nap_3PO in $CDCl_3$ at 30°C

Assignments of the NMR spectra of Nap_3PO are given in Table A. The initial assignment of H_8 in the ^{1}H spectrum was made on the basis of it being the highest frequency signal in 1-substituted naphthalenes [1].

Table S2 ^1H and ^{13}C NMR data for Nap_3PO in $CDCl_3$ and CD_3CN at 30°C

^1H and ^{13}C chemical shifts (ppm) and coupling constants (Hz)					
	$CDCl_3$			CD_3CN	
Atom number	H	C		H	C
1		128.9 (d)<br>$^1J_{PC}$ 104			129.1 (d)<br>$^1J_{PC}$ 102
2	7.24 (m)	133.6 (d)<br>$^2J_{PC}$ 13		7.24 (m)	133.7 (d)<br>$^2J_{PC}$ 12
3	7.32 (m)	124.5 (d)<br>$^3J_{PC}$ 14		7.35 (m)	124.8 (d)<br>$^3J_{PC}$ 15
4	8.02 (d)<br>$^3J_{HH}$ 12	133.3 (d)<br>$^4J_{PC}$ 1		8.13 (d)<br>$^3J_{HH}$ 8	133.3 (d)<br>$^4J_{PC}$ 3
4a		134.1 (d)<br>$^3J_{PC}$ 9			134.1 (d)<br>$^3J_{PC}$ 8
5	7.93 (d)<br>$^3J_{HH}$ 8	128.8 (s)		8.00 (d)<br>$^3J_{HH}$	129.1 (s)
6	7.53 m(m)	126.7 (s)		7.57 (m)	126.9 (s)
7	7.46 (m)	127.5 (s)		7.45 (m)	127.2 (s)
8	8.91 (d)<br>$^3J_{HH}$ 11	128.4 (d)<br>$^4J_{PC}$ 6		8.78 (d)<br>$^3J_{HH}$ 8	127.6 (d)<br>$^4J_{PC}$ 5
8a		134.4 (d)<br>$^2J_{PC}$ 8			134.2 (d)<br>$^2J_{PC}$ 9

From this the assignment of the remaining protons and the carbons in the ^{13}C spectrum was achieved using the COSY (to identify adjacent protons) and heteronuclear correlation spectra (to give the directly bonded carbons), shown in Figure B.

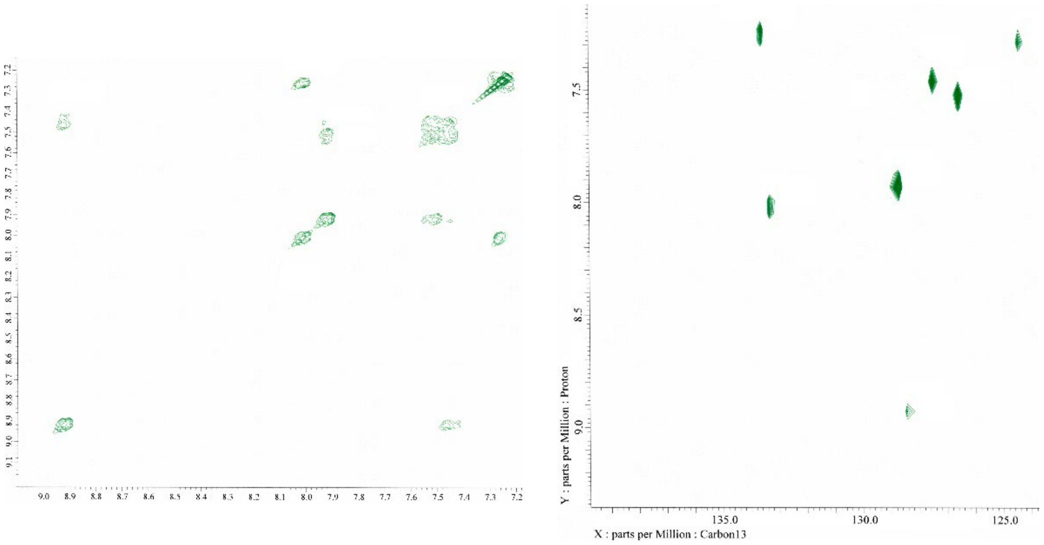

Figure S2 The COSY (left) and the ^{1}H- `^{13}C HETCOR (right) spectra of Nap_3PO in $CDCl_3$ at 30°C

The assignments were confirmed by analysis of the ^{1}H – ^{13}C long range correlation (HMBC) spectrum which is shown in Figure C. In aromatic systems the couplings to the ortho and para carbon atoms are generally small and give low intensity cross peaks or are entirely absent in the spectrum whilst coupling to the carbon meta to the hydrogen is generally in the region of 8 Hz [2] and readily observed in the HMBC spectra. This was particularly useful is assigning C_{4a} and C_{8a}.

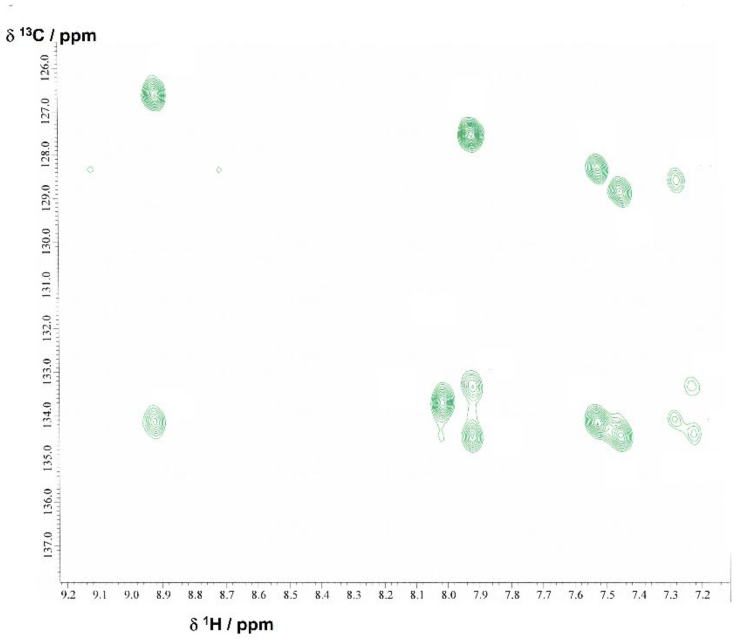

Figure S3 The HMBC spectrum of Nap_3PO in $CDCl_3$ at 30°C

References

1. J.W.Emsley, S.R.Salman, R.A.Storey J.Chem.Soc. (B) 1970, 1513 – 1516

2. M.Barfield, B. Chakrabarti J. Amer. Chem. Soc. 91, (1969), 4346 – 4352

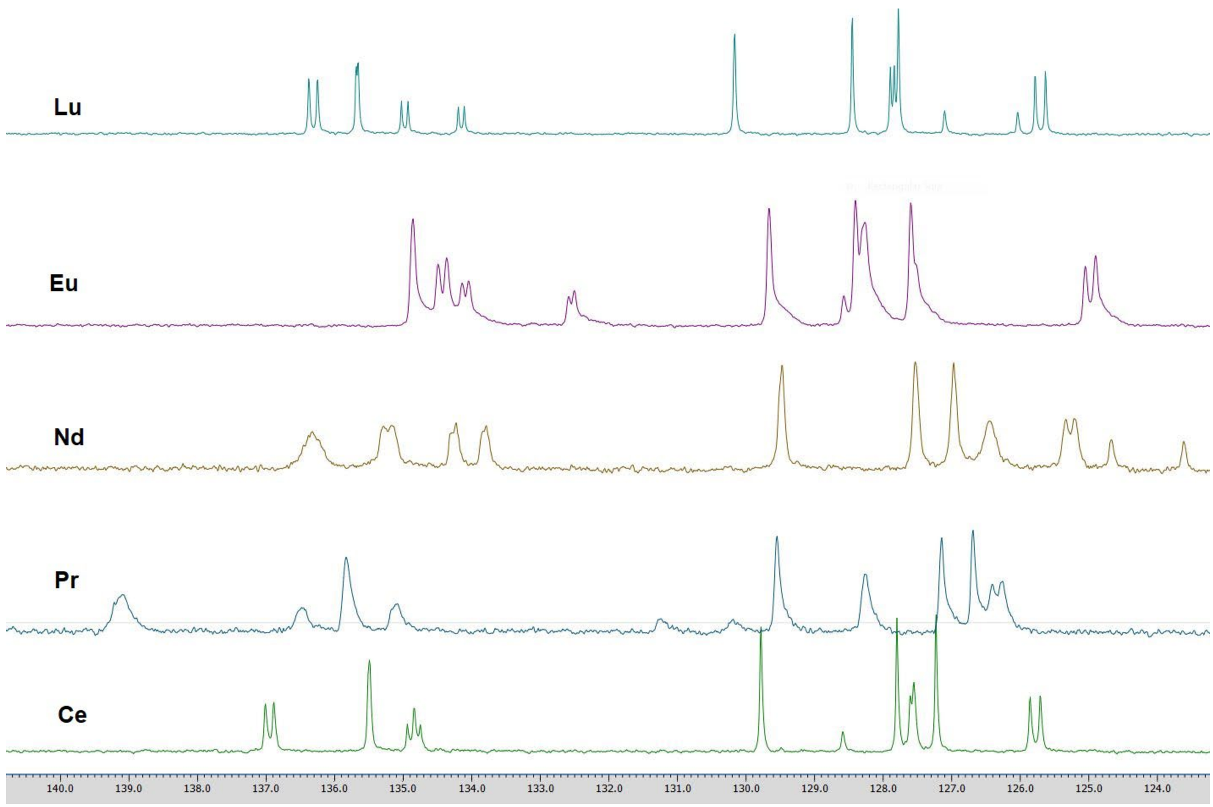

Figure S4 The ^{13}C spectra of the reaction solutions in CD_3CN

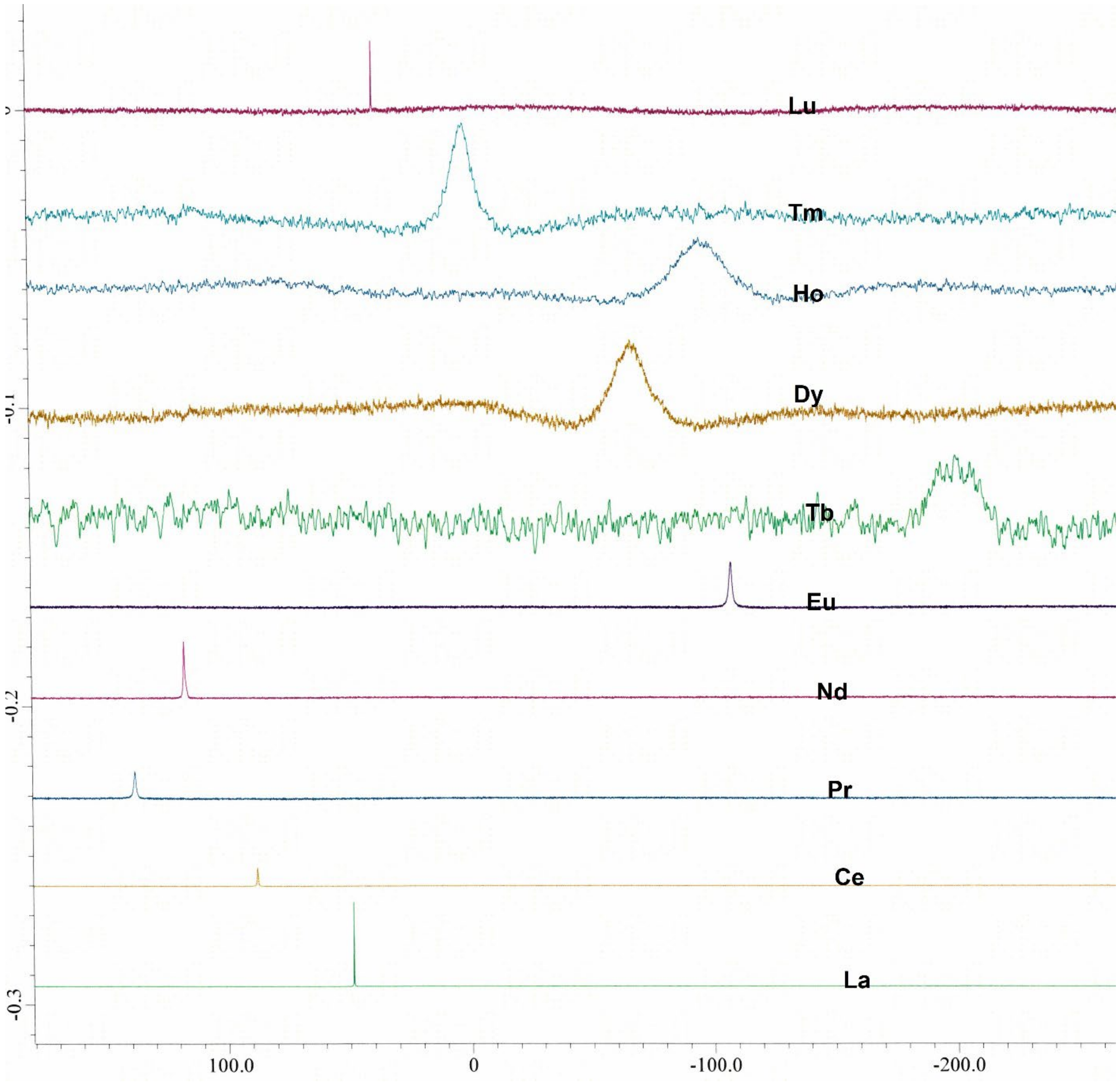

Figure S5 The ^{31}P NMR spectra of the reaction solutions of excess $Ln(NO_3)_3$ with Nap_3PO in CD_3CN at 30°C

Table S3 Mass and volumes of reagents used in the NMR experiments

Mass of reagents (mg) and volume of CD_3CN (ml) used in the NMR experiments

	$Ln(NO_3)_3.6H_2O$	Nap_3PO	Ratio Ln:Nap_3PO	CD_3CN
La	18.0	5.7	3.2:1	0.50
Ce	35.6	5.1	6.9:1	0.52
Ce	5.6	3.7	1.5:1	0.49
Pr	32.5	7.6	4.2:1	0.53
Pr	5.4	9.5	0.6:1	0.50
Nd	19.6	5.7	3.4:1	0.59
Eu	23.0	6.5	3.4:1	0.53
Tb	20.9	6.5	3.0:1	0.48
Dy	28.3	7.0	3.8:	0.56
Ho	33.4	5.0	6.2:1	0.48
Tm	29.2	6.9	3.9:1	0.53

Yb	25.4	5.8	4.0:1	0.73
Yb	6.2	7.6	0.75:1	0.51
Lu	36.7	7.0	4.8:1	0.53

3. Crystallography

Disorder in the structures

The following general procedure was followed in modelling the disorder of the Ln(NO3)3 units. In the initial solution, the Ln centre was located on the inversion centre at , for example 0.5 0.5 0.5 or 0.5 0.5 0, and the nitrogen and oxygen atoms of three nitrate anions were grouped close together such that the nitrate anions appeared to overlap each other. Upon growing the fragments, a series of 6 overlapping nitrate anions could be seen surrounding the central Ln centre. All nitrogen and oxygen atoms of the nitrate anions had larger than ideal isotropic thermal displacement parameters, the thermal ellipsoid of the Ln centre was elongated, and both a large peak and a large hole of electron density could be observed close to the Ln centre. The occupancies of the nitrate anions were lowered to 50%, to maintain charge balance, usually two missing nitrogen atoms could be located in the difference map at this point, and the atoms of some nitrate anions were manually moved to their symmetry equivalent position such that three distinct nitrate anions could be seen in the asymmetric unit. Once the nitrate anions could be resolved, the Ln centre was refined isotropically, and a large peak (ca 20 e/A3) appeared next to the Ln centre. When the structure was grown, this large peak could be seen on both sides of the Ln centre, implying that the Ln centre was also disordered over two symmetry equivalent positions. The original Ln centre was deleted, and the large peak of electron density was assigned as the Ln centre and refined with a fixed occupancy of 50%. This lowered the R value from ca 20% to less than 10%, even with the Ln centre refined anisotropically. The Ln centre was then given anisotropic thermal displacement parameters, and hydrogen atoms were added at their geometrically estimated positions.

$Eu(NO_3)_3(Nap_3PO)_3$

Crystal Data and Experimental

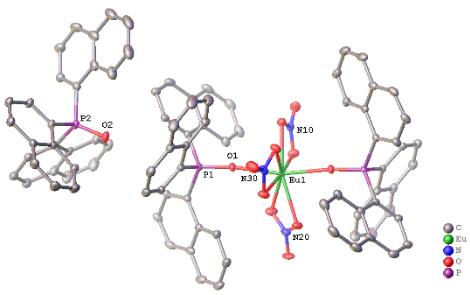

Experimental. Single light yellow irregular-shaped-shaped crystals of **2023NCS0641_1a** were The crystal was chosen from the sample as supplied.. A suitable crystal 0.190×0.110×0.080 mm^3 was selected and mounted on a MITIGEN holder in oil on a Rigaku FRE+ equipped with VHF Varimax confocal mirrors and an UG2 goniometer and HyPix 6000HE detector. The crystal was kept at a steady T = 100.00(10) K during data collection. The structure was solved with the ShelXT 2018/2 (Sheldrick, 2018) structure solution program using the using dual methods solution method and by using Olex2 1.5 (Dolomanov et al., 2009) as the graphical interface. The model was refined with version 2018/3 of ShelXL 2018/3 (Sheldrick, 2015) using full matrix least squares minimisation on $\boldsymbol{F}^2$ minimisation.

Crystal Data. $C_{120}H_{84}N_3O_{13}P_4Eu$, M_r = 2051.74, triclinic, P-1 (No. 2), a = 12.09710(10) Å, b = 13.32270(10) Å, c = 16.31870(10) Å, α =

94.4010(10)°, β = 94.4490(10)°, γ = 115.1360(10)°, V = 2356.19(4) Å^3, T = 100.00(10) K, Z = 1, Z' = 0.5, μ(Mo K$_\alpha$) = 0.804 mm^{-1}, 117232 reflections measured, 14378 unique (R_{int} = 0.0430) which were used in all calculations. The final wR_2 was 0.0708 (all data) and R_1 was 0.0279 (I≥2 σ(I)).

**Compound**	**2023NCS0641_1a**
Formula	$C_{120}H_{84}N_3O_{13}P_4Eu$
$D_{calc.}$/ g cm^{-3}	1.446
μ/mm^{-1}	0.804
Formula Weight	2051.74
Colour	light yellow
Shape	irregular-shaped
Size/mm^3	0.190×0.110×0.080
T/K	100.00(10)
Crystal System	triclinic
Space Group	*P*-1
a/Å	12.09710(10)
b/Å	13.32270(10)
c/Å	16.31870(10)
α/°	94.4010(10)
β/°	94.4490(10)
γ/°	115.1360(10)
V/Å^3	2356.19(4)
Z	1
Z'	0.5
Wavelength/Å	0.71073
Radiation type	Mo K$_\alpha$
Θ_{min}/°	1.701
Θ_{max}/°	30.508
Measured Refl's.	117232
Indep't Refl's	14378
Refl's I≥2 σ(I)	12777
$R_{\rm int}$	0.0430
Parameters	694
Restraints	0
Largest Peak	0.427
Deepest Hole	-0.284
GooF	1.051
wR_2 (all data)	0.0708
wR_2	0.0689
R_1 (all data)	0.0331
R_1	0.0279

Structure Quality Indicators

**Reflections:**	d min (MoKα) 2Θ=61.0° 0.70	I/σ(I) 41.5	Rint m=8.15 4.30%	Full 50.5° 100
**Refinement:**	Shift 0.002	Max Peak 0.4	Min Peak -0.3	GooF 1.051

A light yellow irregular-shaped-shaped crystal with dimensions 0.190×0.110×0.080 mm^3 was mounted on a MITIGEN holder in oil. X-ray diffraction data were collected using a Rigaku FRE+ equipped with VHF Varimax confocal mirrors and an UG2 goniometer and HyPix 6000HE detector equipped with an Oxford Cryosystems low-temperature device, operating at T = 100.00(10) K.

Data were measured using profile data from ω-scans of 0.5 ° per frame for 0.4 s using Mo K$_\alpha$ radiation (Rotating-anode X-ray tube, 45.0 kV, 55.0 mA). The total number of runs and images was based on the strategy calculation from the program CrysAlisPro 1.171.42.99a (Rigaku OD, 2023).The maximum resolution achieved was Θ = 30.508°.

Cell parameters were retrieved using the CrysAlisPro 1.171.42.99a (Rigaku OD, 2023) software and refined

using CrysAlisPro 1.171.42.99a (Rigaku OD, 2023) on 70293 reflections, 60 % of the observed reflections. Data reduction was performed using the CrysAlisPro 1.171.42.99a (Rigaku OD, 2023) software which corrects for Lorentz polarisation. The final completeness is 100.00 % out to 30.508° in Θ.

A analytical absorption correction was performed using CrysAlisPro 1.171.42.99a (Rigaku Oxford Diffraction, 2023) Analytical numeric absorption correction using a multifaceted crystal model based on expressions derived by R.C. Clark & J.S. Reid. (Clark, R. C. & Reid, J. S. (1995). Acta Cryst. A51, 887-897) Empirical absorption correction using spherical harmonics, implemented in SCALE3 ABSPACK scaling algorithm.. The absorption coefficient μ of this material is 0.804 mm^{-1} at this wavelength (λ = 0.71073Å) and the minimum and maximum transmissions are 0.901 and 0.955.

The structure was solved in the space group *P*-1 (# 2) by using dual methods using the ShelXT 2018/2 (Sheldrick, 2018) structure solution program and refined by full matrix least squares minimisation on $\boldsymbol{F^2}$ using version 2018/3 of ShelXL 2018/3 (Sheldrick, 2015). All non-hydrogen atoms were refined anisotropically. Hydrogen atom positions were calculated geometrically and refined using the riding model.

_refine_special_details: The Eu centre and the three nitrate anions are disordered over two symmetry-related sites. Their occupancies have been set at 0.5.

_exptl_absorpt_process_details: CrysAlisPro 1.171.42.99a (Rigaku Oxford Diffraction, 2023)Analytical numeric absorption correction using a multifaceted crystalmodel based on expressions derived by R.C. Clark & J.S. Reid.(Clark, R. C. & Reid, J. S. (1995). Acta Cryst. A51, 887-897) using spherical harmonics,implemented in SCALE3 ABSPACK scaling algorithm.

The value of Z' is 0.5. This means that only half of the formula unit is present in the asymmetric unit, with the other half consisting of symmetry equivalent atoms. The moiety formula is C60 H42 Eu N3 O11 P2, 2(C30 H21 O P).

Table S4: Bond Lengths in Å for **2023NCS0641_1a**.

Atom	Atom	Length/Å
Eu1	O1	2.2699(9)
Eu1	O11	2.4412(18)
Eu1	O12	2.447(2)
Eu1	O21	2.4371(18)
Eu1	O22	2.4698(18)
Eu1	O31	2.473(2)
Eu1	O32	2.4417(18)
Eu1	N10	2.869(2)
Eu1	N20	2.883(2)
Eu1	N30	2.873(3)
P1	O1	1.5144(9)
P1	C1	1.8025(12)
P1	C11	1.8029(12)
P1	C21	1.7972(12)
O11	N10	1.286(3)
O12	N10	1.271(3)
O13	N10	1.213(3)
O21	N20	1.270(3)
O22	N20	1.279(3)
O23	N20	1.197(3)
O31	N30	1.266(3)
O32	N30	1.284(3)
O33	N30	1.217(3)
C1	C2	1.3842(16)
C1	C10	1.4422(16)

Atom	Atom	Length/Å
C2	C3	1.4110(17)
C3	C4	1.3701(19)
C4	C5	1.4174(19)
C5	C6	1.4215(18)
C5	C10	1.4277(17)
C6	C7	1.364(2)
C7	C8	1.409(2)
C8	C9	1.3767(18)
C9	C10	1.4203(18)
C11	C12	1.3770(17)
C11	C20	1.4398(16)
C12	C13	1.4160(17)
C13	C14	1.3605(19)
C14	C15	1.416(2)
C15	C16	1.4175(18)
C15	C20	1.4272(17)
C16	C17	1.364(2)
C17	C18	1.412(2)
C18	C19	1.3745(18)
C19	C20	1.4187(18)
C21	C22	1.3800(17)
C21	C30	1.4369(16)
C22	C23	1.4092(17)
C23	C24	1.365(2)
C24	C25	1.416(2)

Atom	Atom	Length/Å
C25	C26	1.4193(19)
C25	C30	1.4240(17)
C26	C27	1.362(2)
C27	C28	1.408(2)
C28	C29	1.3756(18)
C29	C30	1.4198(18)
P2	O2	1.4872(9)
P2	C31	1.8190(12)
P2	C41	1.8113(13)
P2	C51	1.8219(13)
C31	C32	1.3822(16)
C31	C40	1.4353(16)
C32	C33	1.4098(17)
C33	C34	1.3632(18)
C34	C35	1.4197(17)
C35	C36	1.4180(17)
C35	C40	1.4243(16)
C36	C37	1.3667(19)
C37	C38	1.407(2)
C38	C39	1.3725(18)
C39	C40	1.4212(16)
C41	C42	1.3782(18)

Atom	Atom	Length/Å
C41	C50	1.4393(18)
C42	C43	1.4114(18)
C43	C44	1.369(2)
C44	C45	1.419(2)
C45	C46	1.426(2)
C45	C50	1.424(2)
C46	C47	1.369(3)
C47	C48	1.402(3)
C48	C49	1.371(2)
C49	C50	1.427(2)
C51	C52	1.3796(17)
C51	C60	1.4388(17)
C52	C53	1.4124(18)
C53	C54	1.3696(19)
C54	C55	1.418(2)
C55	C56	1.4214(19)
C55	C60	1.4248(18)
C56	C57	1.366(2)
C57	C58	1.408(2)
C58	C59	1.3758(18)
C59	C60	1.4181(18)

Table S5: Bond Angles in ° for **2023NCS0641_1a**.

Atom	Atom	Atom	Angle/°
O1	Eu1	O11	81.71(5)
O1	Eu1	O12	96.67(7)
O1	Eu1	O21	82.04(5)
O1	Eu1	O22	114.25(5)
O1	Eu1	O31	76.82(7)
O1	Eu1	O32	83.02(5)
O1	Eu1	N10	89.96(5)
O1	Eu1	N20	99.26(5)
O1	Eu1	N30	76.36(5)
O11	Eu1	O12	52.61(9)
O11	Eu1	O22	157.37(6)
O11	Eu1	O31	127.16(8)
O11	Eu1	O32	77.79(6)
O11	Eu1	N10	26.49(7)
O11	Eu1	N20	141.11(6)
O11	Eu1	N30	102.03(7)
O12	Eu1	O22	107.50(9)
O12	Eu1	O31	173.20(9)
O12	Eu1	N10	26.15(8)
O12	Eu1	N20	88.97(9)
O12	Eu1	N30	154.63(7)
O21	Eu1	O11	119.17(6)
O21	Eu1	O12	71.89(9)
O21	Eu1	O22	52.14(7)
O21	Eu1	O31	104.91(8)
O21	Eu1	O32	155.25(6)
O21	Eu1	N10	95.86(7)
O21	Eu1	N20	25.93(7)
O21	Eu1	N30	129.68(7)
O22	Eu1	O31	73.98(9)
O22	Eu1	N10	132.64(7)
O22	Eu1	N20	26.23(7)
O22	Eu1	N30	97.50(8)

Atom	Atom	Atom	Angle/°
O31	Eu1	N10	153.34(7)
O31	Eu1	N20	90.16(8)
O31	Eu1	N30	26.04(7)
O32	Eu1	O12	129.59(9)
O32	Eu1	O22	118.50(7)
O32	Eu1	O31	52.24(8)
O32	Eu1	N10	103.76(7)
O32	Eu1	N20	141.09(6)
O32	Eu1	N30	26.40(7)
N10	Eu1	N20	115.04(7)
N10	Eu1	N30	128.51(7)
N30	Eu1	N20	116.08(7)
O1	P1	C1	110.70(5)
O1	P1	C11	110.73(5)
O1	P1	C21	111.53(5)
C1	P1	C11	107.65(5)
C21	P1	C1	108.59(5)
C21	P1	C11	107.49(5)
P1	O1	Eu1	172.23(5)
N10	O11	Eu1	95.65(15)
N10	O12	Eu1	95.79(17)
N20	O21	Eu1	97.04(14)
N20	O22	Eu1	95.22(14)
N30	O31	Eu1	94.90(17)
N30	O32	Eu1	95.89(16)
O11	N10	Eu1	57.86(12)
O12	N10	Eu1	58.05(14)
O12	N10	O11	115.8(2)
O13	N10	Eu1	177.17(18)
O13	N10	O11	121.8(3)
O13	N10	O12	122.3(2)
O21	N20	Eu1	57.03(12)
O21	N20	O22	115.5(2)

Atom	Atom	Atom	Angle/°
O22	N20	Eu1	58.55(11)
O23	N20	Eu1	177.23(18)
O23	N20	O21	123.2(2)
O23	N20	O22	121.3(2)
O31	N30	Eu1	59.05(15)
O31	N30	O32	116.1(2)
O32	N30	Eu1	57.71(13)
O33	N30	Eu1	171.3(2)
O33	N30	O31	122.3(2)
O33	N30	O32	121.6(3)
C2	C1	P1	118.73(9)
C2	C1	C10	120.14(11)
C10	C1	P1	121.11(9)
C1	C2	C3	120.91(12)
C4	C3	C2	120.20(12)
C3	C4	C5	120.86(12)
C4	C5	C6	120.70(12)
C4	C5	C10	119.94(11)
C6	C5	C10	119.36(12)
C7	C6	C5	121.02(13)
C6	C7	C8	119.86(13)
C9	C8	C7	120.85(13)
C8	C9	C10	120.76(12)
C5	C10	C1	117.93(11)
C9	C10	C1	123.93(11)
C9	C10	C5	118.13(11)
C12	C11	P1	118.08(9)
C12	C11	C20	119.83(11)
C20	C11	P1	122.09(9)
C11	C12	C13	121.32(11)
C14	C13	C12	119.69(12)
C13	C14	C15	121.15(12)
C14	C15	C16	120.99(12)
C14	C15	C20	119.79(11)
C16	C15	C20	119.15(13)
C17	C16	C15	120.99(13)
C16	C17	C18	120.10(12)
C19	C18	C17	120.52(14)
C18	C19	C20	120.73(13)
C15	C20	C11	117.91(11)
C19	C20	C11	123.59(11)
C19	C20	C15	118.45(11)
C22	C21	P1	118.18(9)
C22	C21	C30	119.97(11)
C30	C21	P1	121.85(9)
C21	C22	C23	121.26(12)
C24	C23	C22	119.88(12)
C23	C24	C25	120.91(12)
C24	C25	C26	120.73(12)
C24	C25	C30	119.97(12)
C26	C25	C30	119.28(13)
C27	C26	C25	120.72(13)
C26	C27	C28	120.41(13)
C29	C28	C27	120.49(14)
C28	C29	C30	120.58(13)
C25	C30	C21	117.96(11)
C29	C30	C21	123.53(11)
C29	C30	C25	118.48(11)
O2	P2	C31	115.38(5)
O2	P2	C41	112.99(6)
O2	P2	C51	111.69(5)

Atom	Atom	Atom	Angle/°
C31	P2	C51	104.55(6)
C41	P2	C31	103.84(5)
C41	P2	C51	107.64(6)
C32	C31	P2	117.85(9)
C32	C31	C40	119.38(11)
C40	C31	P2	122.69(8)
C31	C32	C33	121.65(11)
C34	C33	C32	119.93(11)
C33	C34	C35	120.67(11)
C34	C35	C40	119.94(11)
C36	C35	C34	120.36(11)
C36	C35	C40	119.68(11)
C37	C36	C35	120.84(12)
C36	C37	C38	119.58(12)
C39	C38	C37	121.32(12)
C38	C39	C40	120.47(12)
C35	C40	C31	118.37(10)
C39	C40	C31	123.56(11)
C39	C40	C35	118.07(11)
C42	C41	P2	119.89(10)
C42	C41	C50	120.02(12)
C50	C41	P2	120.08(10)
C41	C42	C43	121.15(13)
C44	C43	C42	119.91(13)
C43	C44	C45	121.03(13)
C44	C45	C46	121.37(14)
C44	C45	C50	119.57(13)
C50	C45	C46	119.06(15)
C47	C46	C45	120.96(16)
C46	C47	C48	119.94(15)
C49	C48	C47	121.07(17)
C48	C49	C50	120.69(16)
C45	C50	C41	118.31(13)
C45	C50	C49	118.28(13)
C49	C50	C41	123.41(13)
C52	C51	P2	120.72(9)
C52	C51	C60	119.20(11)
C60	C51	P2	119.81(9)
C51	C52	C53	121.63(12)
C54	C53	C52	119.96(13)
C53	C54	C55	120.71(12)
C54	C55	C56	121.10(13)
C54	C55	C60	119.65(12)
C56	C55	C60	119.23(13)
C57	C56	C55	120.90(13)
C56	C57	C58	120.06(13)
C59	C58	C57	120.62(14)
C58	C59	C60	120.82(13)
C55	C60	C51	118.82(12)
C59	C60	C51	122.80(12)
C59	C60	C55	118.37(11)

Dy(NO3)3(Nap3PO)3

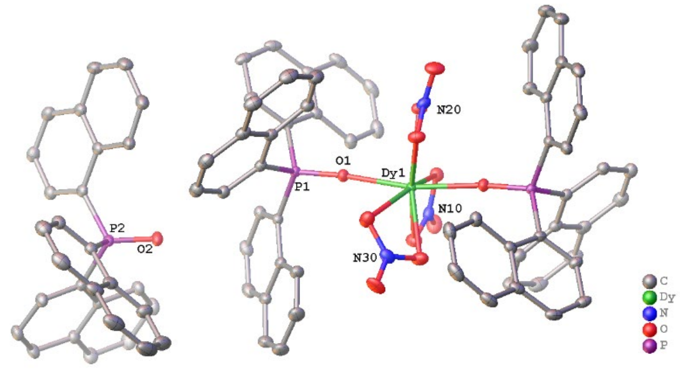

Experimental. Single pale yellow prism-shaped-shaped crystals of **2023NCS0626_1a** were The crystal was chosen from the sample as supplied.. A suitable crystal 0.200×0.190×0.140 mm^3 was selected and mounted on a MITIGEN holder in oil on a Rigaku FRE+ equipped with VHF Varimax confocal mirrors and a UG2 goniometer and HyPix 6000 detector. The crystal was kept at a steady T = 100(2) K during data collection. The structure was solved with the ShelXT 2018/2 (Sheldrick, 2018) structure solution program using the using dual methods solution method and by using Olex2 1.5 (Dolomanov et al., 2009) as the graphical interface. The model was refined with version 2018/3 of ShelXL 2018/3 (Sheldrick, 2015) using full matrix least squares minimisation on $\boldsymbol{F}^2$ minimisation.

Crystal Data. $C_{120}H_{84}N_3O_{13}P_4Dy$, M_r = 2062.28, triclinic, P-1 (No. 2), a = 12.07920(10) Å, b = 13.31390(10) Å, c = 16.31190(10) Å, α = 94.4320(10)°, β = 94.5820(10)°, γ = 115.1890(10)°, V = 2348.18(3) Å^3, T = 100(2) K, Z = 1, Z' = 0.5, μ(Mo K$_\alpha$) = 0.935 mm^{-1}, 116801 reflections measured, 14325 unique (R_{int} = 0.0360) which were used in all calculations. The final wR_2 was 0.0717 (all data) and R_1 was 0.0279 (I≥2 σ(I)).

**Compound**	**2023NCS0626_1a**
Formula	$C_{120}H_{84}N_3O_{13}P_4Dy$
$D_{calc.}$/ g cm^{-3}	1.458
μ/mm^{-1}	0.935
Formula Weight	2062.28
Colour	pale yellow
Shape	prism-shaped
Size/mm^3	0.200×0.190×0.140
T/K	100(2)
Crystal System	triclinic
Space Group	P-1
a/Å	12.07920(10)
b/Å	13.31390(10)
c/Å	16.31190(10)
α/°	94.4320(10)
β/°	94.5820(10)
γ/°	115.1890(10)
V/Å^3	2348.18(3)
Z	1
Z'	0.5
Wavelength/Å	0.71073
Radiation type	Mo K$_\alpha$
Θ_{min}/°	1.703
Θ_{max}/°	30.507
Measured Refl's.	116801
Indep't Refl's	14325
Refl's I≥2 σ(I)	12836
R_{int}	0.0360
Parameters	694
Restraints	0
Largest Peak	0.515
Deepest Hole	-0.253
GooF	1.042
wR_2 (all data)	0.0717
wR_2	0.0699
R_1 (all data)	0.0327
R_1	0.0279

Structure Quality Indicators

**Reflections:**	d min (MoKα) 2Θ=61.0° 0.70	I/σ(I) 47.3	Rint m=8.15 3.60%	Full 50.5° 100
**Refinement:**	Shift 0.001	Max Peak 0.5	Min Peak -0.2	GooF 1.042

A pale yellow prism-shaped-shaped crystal with dimensions 0.200×0.190×0.140 mm^3 was mounted on a MITIGEN holder in oil. X-ray diffraction data were collected using a Rigaku FRE+ equipped with VHF Varimax confocal mirrors and a UG2 goniometer and HyPix 6000 detector equipped with an Oxford Cryosystems low-temperature device, operating at T = 100(2) K.

Data were measured using profile data from ω-scans of 0.5 ° per frame for 0.2 s using Mo K$_\alpha$ radiation

(Rotating Anode, 45.0 kV, 55.0 mA). The total number of runs and images was based on the strategy calculation from the program CrysAlisPro 1.171.42.99a (Rigaku OD, 2023).The maximum resolution achieved was Θ = 30.507°.

Cell parameters were retrieved using the CrysAlisPro 1.171.42.99a (Rigaku OD, 2023) software and refined using CrysAlisPro 1.171.42.99a (Rigaku OD, 2023) on 70833 reflections, 61 % of the observed reflections. Data reduction was performed using the CrysAlisPro 1.171.42.99a (Rigaku OD, 2023) software which corrects for Lorentz polarisation. The final completeness is 100.00 % out to 30.507° in Θ.

A analytical absorption correction was performed using CrysAlisPro 1.171.42.99a (Rigaku Oxford Diffraction, 2023) Analytical numeric absorption correction using a multifaceted crystal model based on expressions derived by R.C. Clark & J.S. Reid. (Clark, R. C. & Reid, J. S. (1995). Acta Cryst. A51, 887-897) Empirical absorption correction using spherical harmonics, implemented in SCALE3 ABSPACK scaling algorithm.. The absorption coefficient μ of this material is 0.935 mm^{-1} at this wavelength (λ = 0.71073Å) and the minimum and maximum transmissions are 0.883 and 0.915.

The structure was solved in the space group *P*-1 (# 2) by using dual methods using the ShelXT 2018/2 (Sheldrick, 2018) structure solution program and refined by full matrix least squares minimisation on $\boldsymbol{F^2}$ using version 2018/3 of ShelXL 2018/3 (Sheldrick, 2015). All non-hydrogen atoms were refined anisotropically. Hydrogen atom positions were calculated geometrically and refined using the riding model.

_refine_special_details: The Dy centre and the three nitrate anions are disordered over two symmetry-related sites.

_exptl_absorpt_process_details: CrysAlisPro 1.171.42.99a (Rigaku Oxford Diffraction, 2023)Analytical numeric absorption correction using a multifaceted crystalmodel based on expressions derived by R.C. Clark & J.S. Reid.(Clark, R. C. & Reid, J. S. (1995). Acta Cryst. A51, 887-897) using spherical harmonics,implemented in SCALE3 ABSPACK scaling algorithm.

The value of Z' is 0.5. This means that only half of the formula unit is present in the asymmetric unit, with the other half consisting of symmetry equivalent atoms. The moiety formula is C60 H42 Dy N3 O11 P2, 2(C30 H21 O P).

Table S6: Bond Lengths in Å for **2023NCS0626_1a**.

Atom	Atom	Length/Å
Dy1	O1	2.2346(9)
Dy1	O11	2.4034(18)
Dy1	O12	2.416(2)
Dy1	O21	2.435(2)
Dy1	O22	2.4035(18)
Dy1	O31	2.4034(19)
Dy1	O32	2.4385(19)
Dy1	N10	2.830(3)
Dy1	N20	2.834(3)
Dy1	N30	2.848(2)
P1	O1	1.5150(9)
P1	C1	1.7978(12)
P1	C11	1.8042(12)
P1	C21	1.8025(12)
O11	N10	1.282(3)
O12	N10	1.270(4)
O13	N10	1.214(3)
O21	N20	1.262(3)
O22	N20	1.284(3)
O23	N20	1.217(3)
O31	N30	1.273(3)
O32	N30	1.278(3)
O33	N30	1.194(3)
C1	C2	1.3783(17)
C1	C10	1.4381(17)
C2	C3	1.4120(17)
C3	C4	1.363(2)
C4	C5	1.416(2)
C5	C6	1.4186(19)
C5	C10	1.4247(17)
C6	C7	1.362(2)
C7	C8	1.412(2)
C8	C9	1.3747(19)
C9	C10	1.4202(19)
C11	C12	1.3778(17)
C11	C20	1.4386(17)
C12	C13	1.4158(18)
C13	C14	1.361(2)

Atom	Atom	Length/Å
C14	C15	1.419(2)
C15	C16	1.4180(19)
C15	C20	1.4277(17)
C16	C17	1.362(2)
C17	C18	1.410(2)
C18	C19	1.3759(19)
C19	C20	1.4172(18)
C21	C22	1.3850(17)
C21	C30	1.4416(17)
C22	C23	1.4127(18)
C23	C24	1.3687(19)
C24	C25	1.4183(19)
C25	C26	1.4234(19)
C25	C30	1.4272(18)
C26	C27	1.366(2)
C27	C28	1.409(2)
C28	C29	1.3768(18)
C29	C30	1.4209(18)
P2	O2	1.4867(9)
P2	C31	1.8120(13)
P2	C41	1.8207(12)
P2	C51	1.8225(13)
C31	C32	1.3765(18)
C31	C40	1.4387(18)
C32	C33	1.4107(19)
C33	C34	1.369(2)
C34	C35	1.419(2)
C35	C36	1.427(2)
C35	C40	1.423(2)
C36	C37	1.369(3)
C37	C38	1.397(3)
C38	C39	1.374(2)
C39	C40	1.426(2)
C41	C42	1.3809(16)
C41	C50	1.4341(17)
C42	C43	1.4124(18)
C43	C44	1.3656(19)
C44	C45	1.4179(17)
C45	C46	1.4180(18)
C45	C50	1.4274(16)
C46	C47	1.366(2)
C47	C48	1.407(2)
C48	C49	1.3731(19)
C49	C50	1.4212(17)
C51	C52	1.3783(18)
C51	C60	1.4413(17)
C52	C53	1.4148(19)
C53	C54	1.3680(19)
C54	C55	1.418(2)
C55	C56	1.4222(19)
C55	C60	1.4246(18)
C56	C57	1.366(2)
C57	C58	1.407(2)
C58	C59	1.3740(18)
C59	C60	1.4165(18)

Table S7: Bond Angles in ° for **2023NCS0626_1a**.

Atom	Atom	Atom	Angle/°
O1	Dy1	O11	87.54(5)
O1	Dy1	O12	83.38(7)
O1	Dy1	O21	102.90(7)
O1	Dy1	O22	85.70(5)
O1	Dy1	O31	112.27(6)
O1	Dy1	O32	78.17(5)
O1	Dy1	N10	84.13(5)
O1	Dy1	N20	97.07(6)
O1	Dy1	N30	95.01(5)
O11	Dy1	O12	53.33(9)
O11	Dy1	O21	127.44(9)
O11	Dy1	O22	77.32(6)
O11	Dy1	O32	157.23(7)
O11	Dy1	N10	26.80(7)
O11	Dy1	N20	102.00(8)
O11	Dy1	N30	141.54(6)
O12	Dy1	O21	173.60(9)
O12	Dy1	O32	106.84(9)
O12	Dy1	N10	26.55(8)
O12	Dy1	N20	155.33(7)
O12	Dy1	N30	88.77(10)
O21	Dy1	O32	73.64(9)
O21	Dy1	N10	153.97(7)
O21	Dy1	N20	26.36(8)
O21	Dy1	N30	89.42(9)
O22	Dy1	O12	129.71(9)
O22	Dy1	O21	52.97(9)
O22	Dy1	O32	118.57(7)
O22	Dy1	N10	103.55(7)
O22	Dy1	N20	26.81(7)
O22	Dy1	N30	141.13(7)
O31	Dy1	O11	119.47(7)
O31	Dy1	O12	72.14(10)
O31	Dy1	O21	103.92(9)
O31	Dy1	O22	154.68(7)
O31	Dy1	O32	52.86(7)
O31	Dy1	N10	96.19(7)
O31	Dy1	N20	128.88(8)
O31	Dy1	N30	26.34(7)
O32	Dy1	N10	132.34(8)
O32	Dy1	N20	97.33(8)
O32	Dy1	N30	26.55(7)
N10	Dy1	N20	128.79(8)
N10	Dy1	N30	115.20(7)
N20	Dy1	N30	115.66(8)
O1	P1	C1	111.58(5)
O1	P1	C11	110.94(5)
O1	P1	C21	110.70(5)
C1	P1	C11	107.30(6)
C1	P1	C21	108.61(5)
C21	P1	C11	107.55(6)
P1	O1	Dy1	173.73(6)
N10	O11	Dy1	95.49(16)
N10	O12	Dy1	95.24(18)

Atom	Atom	Atom	Angle/°
N20	O21	Dy1	94.74(19)
N20	O22	Dy1	95.61(17)
N30	O31	Dy1	96.74(15)
N30	O32	Dy1	94.92(14)
O11	N10	Dy1	57.71(13)
O12	N10	Dy1	58.21(15)
O12	N10	O11	115.9(2)
O13	N10	Dy1	177.29(19)
O13	N10	O11	121.9(3)
O13	N10	O12	122.2(2)
O21	N20	Dy1	58.90(15)
O21	N20	O22	115.8(3)
O22	N20	Dy1	57.58(13)
O23	N20	Dy1	171.9(2)
O23	N20	O21	122.5(3)
O23	N20	O22	121.7(3)
O31	N30	Dy1	56.92(12)
O31	N30	O32	115.4(2)
O32	N30	Dy1	58.53(12)
O33	N30	Dy1	177.19(18)
O33	N30	O31	123.4(2)
O33	N30	O32	121.2(2)
C2	C1	P1	118.08(9)
C2	C1	C10	119.99(11)
C10	C1	P1	121.93(9)
C1	C2	C3	121.24(12)
C4	C3	C2	119.88(13)
C3	C4	C5	120.96(12)
C4	C5	C6	120.75(13)
C4	C5	C10	119.94(12)
C6	C5	C10	119.29(13)
C7	C6	C5	120.70(14)
C6	C7	C8	120.49(13)
C9	C8	C7	120.31(14)
C8	C9	C10	120.65(13)
C5	C10	C1	117.95(12)
C9	C10	C1	123.49(11)
C9	C10	C5	118.53(11)
C12	C11	P1	117.94(9)
C12	C11	C20	119.93(11)
C20	C11	P1	122.13(9)
C11	C12	C13	121.22(12)
C14	C13	C12	119.81(12)
C13	C14	C15	121.04(12)
C14	C15	C20	119.75(12)
C16	C15	C14	120.91(12)
C16	C15	C20	119.25(13)
C17	C16	C15	120.84(13)
C16	C17	C18	120.27(13)
C19	C18	C17	120.49(14)
C18	C19	C20	120.70(13)
C15	C20	C11	117.91(12)
C19	C20	C11	123.65(11)
C19	C20	C15	118.40(11)
C22	C21	P1	118.66(9)
C22	C21	C30	120.10(11)
C30	C21	P1	121.21(9)
C21	C22	C23	120.82(12)
C24	C23	C22	120.32(12)
C23	C24	C25	120.76(12)
C24	C25	C26	120.55(13)

Atom	Atom	Atom	Angle/°
C24	C25	C30	119.98(12)
C26	C25	C30	119.47(12)
C27	C26	C25	120.89(13)
C26	C27	C28	119.80(13)
C29	C28	C27	121.03(13)
C28	C29	C30	120.67(13)
C25	C30	C21	118.00(11)
C29	C30	C21	123.87(12)
C29	C30	C25	118.12(11)
O2	P2	C31	113.00(6)
O2	P2	C41	115.45(6)
O2	P2	C51	111.74(6)
C31	P2	C41	103.80(6)
C31	P2	C51	107.62(6)
C41	P2	C51	104.44(6)
C32	C31	P2	119.98(10)
C32	C31	C40	119.95(12)
C40	C31	P2	120.06(10)
C31	C32	C33	121.37(13)
C34	C33	C32	119.73(14)
C33	C34	C35	121.04(13)
C34	C35	C36	121.35(15)
C34	C35	C40	119.64(13)
C40	C35	C36	119.00(15)
C37	C36	C35	120.92(16)
C36	C37	C38	120.06(15)
C39	C38	C37	121.06(17)
C38	C39	C40	120.65(16)
C35	C40	C31	118.27(13)
C35	C40	C39	118.30(13)
C39	C40	C31	123.43(13)
C42	C41	P2	117.76(9)
C42	C41	C50	119.61(11)
C50	C41	P2	122.56(9)
C41	C42	C43	121.52(12)
C44	C43	C42	119.90(12)
C43	C44	C45	120.71(12)
C44	C45	C46	120.46(11)
C44	C45	C50	119.93(11)
C46	C45	C50	119.61(11)
C47	C46	C45	120.91(12)
C46	C47	C48	119.58(13)
C49	C48	C47	121.37(12)
C48	C49	C50	120.47(12)
C45	C50	C41	118.30(11)
C49	C50	C41	123.67(11)
C49	C50	C45	118.03(11)
C52	C51	P2	120.69(9)
C52	C51	C60	119.21(12)
C60	C51	P2	119.81(9)
C51	C52	C53	121.57(12)
C54	C53	C52	120.00(13)
C53	C54	C55	120.74(13)
C54	C55	C56	121.14(13)
C54	C55	C60	119.66(12)
C56	C55	C60	119.18(13)
C57	C56	C55	120.83(14)
C56	C57	C58	120.08(13)
C59	C58	C57	120.63(14)
C58	C59	C60	120.88(13)
C55	C60	C51	118.79(12)

Atom	Atom	Atom	Angle/˚
C59	C60	C51	122.80(12)
C59	C60	C55	118.39(11)

$Ho(NO_3)_3(Nap_3PO)_3$

Crystal Data and Experimental

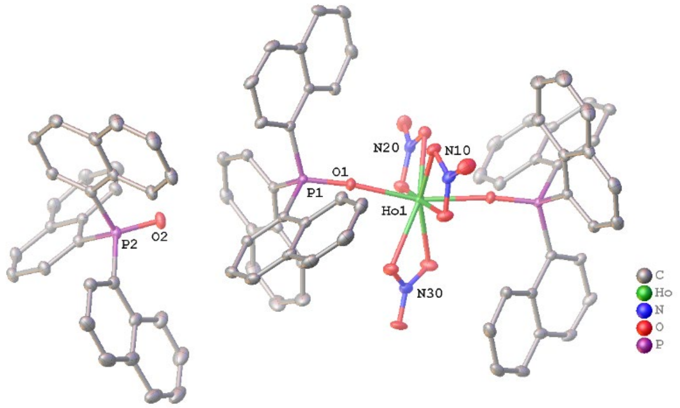

Experimental. Single yellow irregular-shaped-shaped crystals of **2023NCS0640_2a** were chosen from the sample as supplied. A suitable crystal 0.190×0.150×0.120 mm^3 was selected and mounted on a MITIGEN holder in oil on a Rigaku FRE+ equipped with VHF Varimax confocal mirrors and a UG2 goniometer and HyPix 6000 detector. The crystal was kept at a steady T = 100(2) K during data collection. The structure was solved with the ShelXT 2018/2 (Sheldrick, 2015) structure solution program using the using dual methods solution method and by using Olex2 1.5 (Dolomanov et al., 2009) as the graphical interface. The model was refined with version 2018/3 of ShelXL 2018/3 (Sheldrick, 2015) using full matrix least squares minimisation on $\boldsymbol{F}^2$ minimisation.

Crystal Data. $C_{120}H_{84}N_3O_{13}P_4Ho$, M_r = 2064.71, triclinic, P-1 (No. 2), a = 12.07620(10) Å, b = 13.30500(10) Å, c = 16.30450(10) Å, α = 94.4900(10)°, β = 94.5370(10)°, γ = 115.1980(10)°, V = 2344.72(3) Å^3, T = 100(2) K, Z = 1, Z' = 0.5, μ(Mo K$_\alpha$) = 0.983 mm^{-1}, 185205 reflections measured, 17391 unique (R_{int} = 0.0394) which were used in all calculations. The final wR_2 was 0.0700 (all data) and R_1 was 0.0276 (I≥2 σ(I)).

**Compound**	**2023NCS0640_2a**
Formula	$C_{120}H_{84}N_3O_{13}P_4Ho$
$D_{calc.}$/ g cm^{-3}	1.462
μ/mm^{-1}	0.983
Formula Weight	2064.71
Colour	yellow
Shape	irregular-shaped
Size/mm^3	0.190×0.150×0.120
T/K	100(2)
Crystal System	triclinic
Space Group	P-1
a/Å	12.07620(10)
b/Å	13.30500(10)
c/Å	16.30450(10)
α/°	94.4900(10)
β/°	94.5370(10)
γ/°	115.1980(10)
V/Å^3	2344.72(3)
Z	1
Z'	0.5
Wavelength/Å	0.71073
Radiation type	Mo K$_\alpha$
Θ_{min}/°	1.705
Θ_{max}/°	33.648
Measured Refl's.	185205
Indep't Refl's	17391
Refl's I≥2 σ(I)	15397
R_{int}	0.0394
Parameters	694
Restraints	0
Largest Peak	0.447
Deepest Hole	-0.315
GooF	1.034
wR_2 (all data)	0.0700
wR_2	0.0679
R_1 (all data)	0.0337
R_1	0.0276

Structure Quality Indicators

**Reflections:**	d min (MoKα) 2Θ=67.3° 0.64	I/σ(I) 44.8	Rint m=10.65 3.94%	Full 50.5° 93% to 67.3° 100
**Refinement:**	Shift -0.002	Max Peak 0.5	Min Peak -0.3	GooF 1.034

A yellow irregular-shaped crystal with dimensions 0.190×0.150×0.120 mm^3 was mounted on a MITIGEN holder in oil. X-ray diffraction data were collected using a Rigaku FRE+ equipped with VHF Varimax confocal mirrors and a UG2 goniometer and HyPix 6000 detector equipped with an Oxford Cryosystems low-temperature device, operating at T = 100(2) K.

Data were measured using profile data from ω-scans of 0.5 ° per frame for 0.3 s using Mo K_{α} radiation (Rotating-anode X-ray tube, 45.0 kV, 55.0 mA). The total number of runs and images was based on the strategy calculation from the program CrysAlisPro 1.171.42.99a (Rigaku OD, 2023). The maximum resolution achieved was Θ = 33.648°.

Cell parameters were retrieved using the CrysAlisPro 1.171.42.99a (Rigaku OD, 2023) software and refined using CrysAlisPro 1.171.42.99a (Rigaku OD, 2023) on 109513 reflections, 59 % of the observed reflections. Data reduction was performed using the CrysAlisPro 1.171.42.99a (Rigaku OD, 2023) software which corrects for Lorentz polarisation. The final completeness is 100.00 % out to 33.648° in Θ.

An analytical absorption correction was performed using CrysAlisPro 1.171.42.99a (Rigaku Oxford Diffraction, 2023) Analytical numeric absorption correction using a multifaceted crystal model based on expressions derived by R.C. Clark & J.S. Reid. (Clark, R. C. & Reid, J. S. (1995). Acta Cryst. A51, 887-897) Empirical absorption correction using spherical harmonics, implemented in SCALE3 ABSPACK scaling algorithm. The absorption coefficient μ of this material is 0.983 mm^{-1} at this wavelength (λ = 0.71073Å) and the minimum and maximum transmissions are 0.871 and 0.931.

The structure was solved in the space group P-1 (# 2) by using dual methods using the ShelXT 2018/2 (Sheldrick, 2015) structure solution program and refined by full matrix least squares minimisation on $\boldsymbol{F}^2$ using version 2018/3 of ShelXL 2018/3 (Sheldrick, 2015). All non-hydrogen atoms were refined anisotropically. Hydrogen atom positions were calculated geometrically and refined using the riding model.

_refine_special_details: The Ho centre and the three nitrate anions are disordered and have been modelled over two symmetry-related sites, each of 50% occupancy.

_exptl_absorpt_process_details: CrysAlisPro 1.171.42.99a (Rigaku Oxford Diffraction, 2023) Analytical numeric absorption correction using a multifaceted crystal model based on expressions derived by R.C. Clark & J.S. Reid. (Clark, R. C. & Reid, J. S. (1995). Acta Cryst. A51, 887-897) using spherical harmonics, implemented in SCALE3 ABSPACK scaling algorithm.

The value of Z' is 0.5. This means that only half of the formula unit is present in the asymmetric unit, with the other half consisting of symmetry equivalent atoms. The moiety formula is C60 H42 Ho N3 O11 P2, 2(C30 H21 O P).

Table S8: Bond Lengths in Å for **2023NCS0640_2a**.

Atom	Atom	Length/Å
Ho1	O1	2.2283(7)
Ho1	O11	2.3917(15)
Ho1	O12	2.4190(17)
Ho1	O21	2.3962(18)
Ho1	O22	2.3887(15)
Ho1	O31	2.4237(15)
Ho1	O32	2.3903(15)
Ho1	N10	2.817(2)
Ho1	N20	2.814(2)
Ho1	N30	2.8330(17)
P1	O1	1.5125(8)
P1	C1	1.8054(9)
P1	C11	1.7981(10)
P1	C21	1.8053(10)
O11	N10	1.282(3)
O12	N10	1.266(3)
O13	N10	1.221(3)
O21	N20	1.274(3)
O22	N20	1.283(3)
O23	N20	1.215(3)

Atom	Atom	Length/Å
O31	N30	1.277(3)
O32	N30	1.272(2)
O33	N30	1.201(2)
C1	C2	1.3761(14)
C1	C10	1.4409(13)
C2	C3	1.4160(14)
C3	C4	1.3635(17)
C4	C5	1.4153(17)
C5	C6	1.4194(15)
C5	C10	1.4289(14)
C6	C7	1.365(2)
C7	C8	1.4113(18)
C8	C9	1.3769(15)
C9	C10	1.4159(15)
C11	C12	1.3799(14)
C11	C20	1.4375(14)
C12	C13	1.4105(15)
C13	C14	1.3663(17)
C14	C15	1.4144(17)
C15	C16	1.4212(16)
C15	C20	1.4242(15)
C16	C17	1.363(2)
C17	C18	1.410(2)
C18	C19	1.3765(15)
C19	C20	1.4205(15)
C21	C22	1.3848(14)
C21	C30	1.4374(14)
C22	C23	1.4143(14)
C23	C24	1.3680(17)
C24	C25	1.4193(16)
C25	C26	1.4193(16)
C25	C30	1.4298(14)
C26	C27	1.3687(19)
C27	C28	1.4100(18)
C28	C29	1.3763(16)
C29	C30	1.4216(15)
P2	O2	1.4881(8)

Atom	Atom	Length/Å
P2	C31	1.8238(11)
P2	C41	1.8186(11)
P2	C51	1.8118(11)
C31	C32	1.3780(15)
C31	C40	1.4381(15)
C32	C33	1.4162(15)
C33	C34	1.3691(17)
C34	C35	1.4182(17)
C35	C36	1.4192(17)
C35	C40	1.4277(15)
C36	C37	1.366(2)
C37	C38	1.4109(18)
C38	C39	1.3739(16)
C39	C40	1.4154(16)
C41	C42	1.3827(14)
C41	C50	1.4334(14)
C42	C43	1.4087(15)
C43	C44	1.3679(16)
C44	C45	1.4192(15)
C45	C46	1.4184(15)
C45	C50	1.4256(14)
C46	C47	1.3677(16)
C47	C48	1.4071(17)
C48	C49	1.3740(16)
C49	C50	1.4240(14)
C51	C52	1.3763(15)
C51	C60	1.4399(15)
C52	C53	1.4131(15)
C53	C54	1.3688(19)
C54	C55	1.417(2)
C55	C56	1.4273(18)
C55	C60	1.4257(17)
C56	C57	1.369(3)
C57	C58	1.398(2)
C58	C59	1.3764(18)
C59	C60	1.4258(18)

Table S9: Bond Angles in ° for **2023NCS0640_2a**.

Atom	Atom	Atom	Angle/°
O1	Ho1	O11	85.64(4)
O1	Ho1	O12	102.61(5)
O1	Ho1	O21	83.41(5)
O1	Ho1	O22	87.35(4)
O1	Ho1	O31	78.39(5)
O1	Ho1	O32	112.36(5)
O1	Ho1	N10	96.87(5)
O1	Ho1	N20	84.02(4)
O1	Ho1	N30	95.21(4)
O11	Ho1	O12	53.37(7)
O11	Ho1	O21	129.34(8)
O11	Ho1	O31	118.68(6)
O11	Ho1	N10	26.95(6)
O11	Ho1	N20	102.90(6)
O11	Ho1	N30	141.44(5)
O12	Ho1	O31	73.12(8)
O12	Ho1	N10	26.61(6)
O12	Ho1	N20	153.92(6)

Atom	Atom	Atom	Angle/°
O12	Ho1	N30	89.24(8)
O21	Ho1	O12	173.83(7)
O21	Ho1	O31	107.16(8)
O21	Ho1	N10	155.17(6)
O21	Ho1	N20	26.81(7)
O21	Ho1	N30	88.86(8)
O22	Ho1	O11	76.46(5)
O22	Ho1	O12	127.17(8)
O22	Ho1	O21	53.78(8)
O22	Ho1	O31	157.83(6)
O22	Ho1	O32	119.74(6)
O22	Ho1	N10	101.39(6)
O22	Ho1	N20	27.00(6)
O22	Ho1	N30	142.09(5)
O31	Ho1	N10	97.14(7)
O31	Ho1	N20	132.88(7)
O31	Ho1	N30	26.70(6)
O32	Ho1	O11	155.08(6)

Atom	Atom	Atom	Angle/°
O32	Ho1	O12	104.14(7)
O32	Ho1	O21	71.93(8)
O32	Ho1	O31	53.17(6)
O32	Ho1	N10	129.28(6)
O32	Ho1	N20	96.25(6)
O32	Ho1	N30	26.49(6)
N10	Ho1	N30	115.75(6)
N20	Ho1	N10	128.38(6)
N20	Ho1	N30	115.54(6)
O1	P1	C1	110.85(4)
O1	P1	C11	111.67(4)
O1	P1	C21	110.84(4)
C1	P1	C21	107.46(5)
C11	P1	C1	107.28(5)
C11	P1	C21	108.57(5)
P1	O1	Ho1	173.31(5)
N10	O11	Ho1	95.33(14)
N10	O12	Ho1	94.51(15)
N20	O21	Ho1	95.20(15)
N20	O22	Ho1	95.31(13)
N30	O31	Ho1	94.81(12)
N30	O32	Ho1	96.55(12)
O11	N10	Ho1	57.71(11)
O12	N10	Ho1	58.88(12)
O12	N10	O11	116.0(2)
O13	N10	Ho1	171.96(17)
O13	N10	O11	121.7(2)
O13	N10	O12	122.3(2)
O21	N20	Ho1	57.99(12)
O21	N20	O22	115.62(19)
O22	N20	Ho1	57.69(10)
O23	N20	Ho1	177.47(16)
O23	N20	O21	122.0(2)
O23	N20	O22	122.4(2)
O31	N30	Ho1	58.49(10)
O32	N30	Ho1	56.95(10)
O32	N30	O31	115.37(16)
O33	N30	Ho1	177.36(16)
O33	N30	O31	121.4(2)
O33	N30	O32	123.2(2)
C2	C1	P1	117.98(7)
C2	C1	C10	120.03(9)
C10	C1	P1	121.99(7)
C1	C2	C3	121.27(10)
C4	C3	C2	119.68(10)
C3	C4	C5	121.07(9)
C4	C5	C6	120.90(10)
C4	C5	C10	119.94(9)
C6	C5	C10	119.07(11)
C7	C6	C5	120.92(11)
C6	C7	C8	120.17(10)
C9	C8	C7	120.51(11)
C8	C9	C10	120.71(10)
C5	C10	C1	117.67(9)
C9	C10	C1	123.72(9)
C9	C10	C5	118.57(9)
C12	C11	P1	118.14(7)
C12	C11	C20	119.89(9)
C20	C11	P1	121.97(8)
C11	C12	C13	121.24(10)
C14	C13	C12	119.94(10)

Atom	Atom	Atom	Angle/°
C13	C14	C15	120.81(10)
C14	C15	C16	120.67(11)
C14	C15	C20	120.05(10)
C16	C15	C20	119.26(11)
C17	C16	C15	120.71(12)
C16	C17	C18	120.36(11)
C19	C18	C17	120.55(12)
C18	C19	C20	120.48(11)
C15	C20	C11	118.02(10)
C19	C20	C11	123.36(10)
C19	C20	C15	118.60(9)
C22	C21	P1	118.54(8)
C22	C21	C30	120.30(9)
C30	C21	P1	121.14(7)
C21	C22	C23	120.70(10)
C24	C23	C22	120.24(10)
C23	C24	C25	120.94(10)
C24	C25	C26	120.63(10)
C24	C25	C30	119.72(10)
C26	C25	C30	119.65(10)
C27	C26	C25	120.87(11)
C26	C27	C28	119.80(11)
C29	C28	C27	120.91(11)
C28	C29	C30	120.85(10)
C25	C30	C21	118.08(9)
C29	C30	C21	124.02(9)
C29	C30	C25	117.90(10)
O2	P2	C31	111.68(5)
O2	P2	C41	115.42(5)
O2	P2	C51	113.05(5)
C41	P2	C31	104.56(5)
C51	P2	C31	107.61(5)
C51	P2	C41	103.75(5)
C32	C31	P2	120.47(8)
C32	C31	C40	119.43(10)
C40	C31	P2	119.83(8)
C31	C32	C33	121.44(10)
C34	C33	C32	119.96(11)
C33	C34	C35	120.86(10)
C34	C35	C36	121.18(11)
C34	C35	C40	119.47(10)
C36	C35	C40	119.33(11)
C37	C36	C35	120.87(11)
C36	C37	C38	120.01(11)
C39	C38	C37	120.54(12)
C38	C39	C40	121.03(11)
C35	C40	C31	118.81(10)
C39	C40	C31	122.96(10)
C39	C40	C35	118.22(10)
C42	C41	P2	117.88(8)
C42	C41	C50	119.28(9)
C50	C41	P2	122.77(7)
C41	C42	C43	121.84(10)
C44	C43	C42	119.81(10)
C43	C44	C45	120.60(10)
C44	C45	C50	119.91(10)
C46	C45	C44	120.34(10)
C46	C45	C50	119.74(10)
C47	C46	C45	120.80(11)
C46	C47	C48	119.68(11)
C49	C48	C47	121.28(10)

Atom	Atom	Atom	Angle/°
C48	C49	C50	120.46(10)
C45	C50	C41	118.52(9)
C49	C50	C41	123.48(9)
C49	C50	C45	118.00(9)
C52	C51	P2	119.90(8)
C52	C51	C60	120.12(10)
C60	C51	P2	119.97(8)
C51	C52	C53	121.25(11)
C54	C53	C52	119.77(12)
C53	C54	C55	121.04(11)
C54	C55	C56	121.45(12)
C54	C55	C60	119.78(11)
C60	C55	C56	118.77(13)
C57	C56	C55	121.04(14)
C56	C57	C58	120.16(12)
C59	C58	C57	120.93(15)
C58	C59	C60	120.56(13)
C55	C60	C51	118.04(11)
C55	C60	C59	118.54(11)
C59	C60	C51	123.43(11)

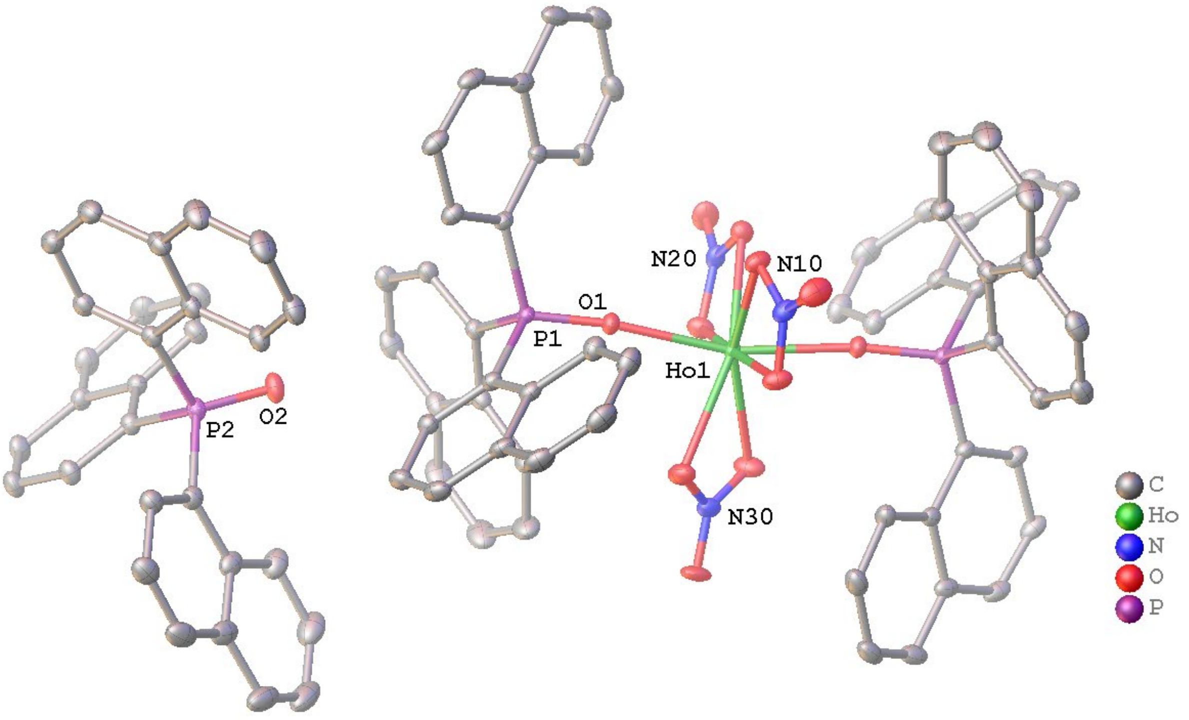
N20
N10
O1
P1
Ho1
P2
O2
N30
C
Ho
N
O
P

$Er(NO_3)_3(Nap_3PO)_3$

Crystal Data and Experimental

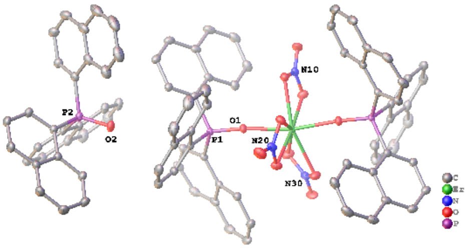

Compound	2023NCS0627_1a
Formula	$C_{120}H_{84}ErN_3O_{13}P_4$
$D_{calc.}$/ g cm^{-3}	1.464
μ/mm^{-1}	1.034
Formula Weight	2067.04
Colour	pale yellow
Shape	irregular-shaped
Size/mm^3	0.34×0.21×0.10
T/K	100(2)
Crystal System	triclinic
Space Group	P-1
a/Å	12.07150(10)
b/Å	13.30700(10)
c/Å	16.30720(10)
α/°	94.5250(10)
β/°	94.5320(10)
γ/°	115.1750(10)
V/Å^3	2344.83(3)
Z	1
Z'	0.5
Wavelength/Å	0.71073
Radiation type	Mo K_α
Θ_{min}/°	1.704
Θ_{max}/°	30.507
Measured Refl's.	332833
Indep't Refl's	14302
Refl's I≥2 σ(I)	13199
R_{int}	0.0685
Parameters	694
Restraints	0
Largest Peak	0.485
Deepest Hole	-0.393
GooF	1.046
wR_2 (all data)	0.0675
wR_2	0.0662
R_1 (all data)	0.0279
R_1	0.0250

Experimental. Single pale yellow irregular-shaped-shaped crystals of **2023NCS0627_1a** were The crystal was chosen from the sample as supplied.. A suitable crystal 0.34×0.21×0.10 mm^3 was selected and mounted on a MITIGEN holder in oil on a Rigaku FRE+ equipped with VHF Varimax confocal mirrors and a UG2 goniometer and HyPix 6000 detector. The crystal was kept at a steady T = 100(2) K during data collection. The structure was solved with the ShelXT 2018/2 (Sheldrick, 2018) structure solution program using the using dual methods solution method and by using Olex2 1.3 (Dolomanov et al., 2009) as the graphical interface. The model was refined with version 2018/3 of ShelXL 2018/3 (Sheldrick, 2015) using full matrix least squares minimisation on $\boldsymbol{F^2}$ minimisation.

Crystal Data. $C_{120}H_{84}ErN_3O_{13}P_4$, M_r = 2067.04, triclinic, P-1 (No. 2), a = 12.07150(10) Å, b = 13.30700(10) Å, c = 16.30720(10) Å, α = 94.5250(10)°, β = 94.5320(10)°, γ = 115.1750(10)°, V = 2344.83(3) Å^3, T = 100(2) K, Z = 1, Z' = 0.5, μ(Mo K_α) = 1.034 mm^{-1}, 332833 reflections measured, 14302 unique (R_{int} = 0.0685) which were used in all calculations. The final wR_2 was 0.0675 (all data) and R_1 was 0.0250 (I≥2 σ(I)).

Structure Quality Indicators

**Reflections:**	d min (Mo) 2θ=61.0° 0.70	I/σ(I) 54.2	Rint 6.85%	CAP 60.9° 100
**Refinement:**	Shift 0.002	Max Peak 0.5	Min Peak -0.4	GooF 1.046

A pale yellow irregular-shaped-shaped crystal with dimensions 0.34×0.21×0.10 mm^3 was mounted on a

MITIGEN holder in oil. X-ray diffraction data were collected using a Rigaku FRE+ equipped with VHF Varimax confocal mirrors and a UG2 goniometer and HyPix 6000 detector equipped with an Oxford Cryosystems low-temperature device, operating at T = 100(2) K.

Data were measured using profile data from ω-scans of ° per frame for s using Mo K_α radiation (Rotating-anode X-ray tube, 45.0 kV, 55.0 mA). The total number of runs and images was based on the strategy calculation from the program CrysAlisPro 1.171.42.99a (Rigaku OD, 2023).The maximum resolution achieved was Θ = 30.507°.

Cell parameters were retrieved using the CrysAlisPro 1.171.42.99a (Rigaku OD, 2023) software and refined using CrysAlisPro 1.171.42.99a (Rigaku OD, 2023) on 196816 reflections, 59 % of the observed reflections. Data reduction was performed using the CrysAlisPro 1.171.42.99a (Rigaku OD, 2023) software which corrects for Lorentz polarisation. The final completeness is 100.00 % out to 30.507° in Θ.

A analytical absorption correction was performed using CrysAlisPro 1.171.42.99a (Rigaku Oxford Diffraction, 2023) Analytical numeric absorption correction using a multifaceted crystal model based on expressions derived by R.C. Clark & J.S. Reid. (Clark, R. C. & Reid, J. S. (1995). Acta Cryst. A51, 887-897) Empirical absorption correction using spherical harmonics, implemented in SCALE3 ABSPACK scaling algorithm.. The absorption coefficient μ of this material is 1.034 mm^{-1} at this wavelength (λ = 0.71073Å) and the minimum and maximum transmissions are 0.783 and 0.927.

The structure was solved in the space group P-1 (# 2) by using dual methods using the ShelXT 2018/2 (Sheldrick, 2018) structure solution program and refined by full matrix least squares minimisation on $\boldsymbol{F^2}$ using version 2018/3 of ShelXL 2018/3 (Sheldrick, 2015). All non-hydrogen atoms were refined anisotropically. Hydrogen atom positions were calculated geometrically and refined using the riding model.

_refine_special_details: The Er centre and all three nitrate anions were found to be disordered, and have been modelled over two symmetry-related sites.

_exptl_absorpt_process_details: CrysAlisPro 1.171.42.99a (Rigaku Oxford Diffraction, 2023)Analytical numeric absorption correction using a multifaceted crystalmodel based on expressions derived by R.C. Clark & J.S. Reid.(Clark, R. C. & Reid, J. S. (1995). Acta Cryst. A51, 887-897)Empirical absorption correction using spherical harmonics,implemented in SCALE3 ABSPACK scaling algorithm.

The value of Z' is 0.5. This means that only half of the formula unit is present in the asymmetric unit, with the other half consisting of symmetry equivalent atoms.

Table S10: Bond Lengths in Å for **2023NCS0627_1a**.

Atom	Atom	Length/Å
Er^1	Er^{11}	0.56267(14)
Er^1	O^{11}	2.2134(8)
Er^1	O^1	2.2188(8)
Er^1	O^{11}	2.3784(16)
Er^1	O^{12}	2.384(2)
Er^1	O^{21}	2.4036(19)
Er^1	O^{22}	2.3792(16)
Er^1	O^{31}	2.3786(17)
Er^1	O^{32}	2.4129(17)
Er^1	N^{10}	2.801(2)
Er^1	N^{20}	2.803(3)
Er^1	N^{30}	2.8215(19)
P^1	O^1	1.5140(8)
P^1	C^1	1.8075(10)

Atom	Atom	Length/Å
P^1	C^{11}	1.8043(11)
P^1	C^{21}	1.7988(11)
O^{11}	N^{10}	1.284(3)
O^{12}	N^{10}	1.271(3)
O^{13}	N^{10}	1.213(3)
O^{21}	N^{20}	1.263(3)
O^{22}	N^{20}	1.284(3)
O^{23}	N^{20}	1.219(3)
O^{31}	N^{30}	1.273(3)
O^{32}	N^{30}	1.273(3)
O^{33}	N^{30}	1.203(2)
C^1	C^2	1.3768(15)
C^1	C^{10}	1.4367(15)
C^2	C^3	1.4161(15)

Atom	Atom	Length/Å
C^{3}	C^{4}	1.3626(18)
C^{4}	C^{5}	1.4138(18)
C^{5}	C^{6}	1.4196(17)
C^{5}	C^{10}	1.4304(15)
C^{6}	C^{7}	1.363(2)
C^{7}	C^{8}	1.411(2)
C^{8}	C^{9}	1.3765(16)
C^{9}	C^{10}	1.4170(16)
C^{11}	C^{12}	1.3853(15)
C^{11}	C^{20}	1.4387(15)
C^{12}	C^{13}	1.4129(15)
C^{13}	C^{14}	1.3676(18)
C^{14}	C^{15}	1.4184(18)
C^{15}	C^{16}	1.4212(17)
C^{15}	C^{20}	1.4291(16)
C^{16}	C^{17}	1.368(2)
C^{17}	C^{18}	1.412(2)
C^{18}	C^{19}	1.3763(17)
C^{19}	C^{20}	1.4247(16)
C^{21}	C^{22}	1.3807(15)
C^{21}	C^{30}	1.4369(15)
C^{22}	C^{23}	1.4121(16)
C^{23}	C^{24}	1.3653(19)
C^{24}	C^{25}	1.4149(19)
C^{25}	C^{26}	1.4209(17)
C^{25}	C^{30}	1.4256(16)
C^{26}	C^{27}	1.362(2)
C^{27}	C^{28}	1.411(2)
C^{28}	C^{29}	1.3769(17)
C^{29}	C^{30}	1.4200(16)
P^{2}	O^{2}	1.4875(9)
P^{2}	C^{31}	1.8239(11)
P^{2}	C^{41}	1.8198(11)
P^{2}	C^{51}	1.8109(12)
C^{31}	C^{32}	1.3781(16)
C^{31}	C^{40}	1.4412(16)
C^{32}	C^{33}	1.4167(17)
C^{33}	C^{34}	1.3684(19)
C^{34}	C^{35}	1.4190(18)
C^{35}	C^{36}	1.4200(18)
C^{35}	C^{40}	1.4262(16)
C^{36}	C^{37}	1.368(2)
C^{37}	C^{38}	1.409(2)
C^{38}	C^{39}	1.3750(17)
C^{39}	C^{40}	1.4156(17)
C^{41}	C^{42}	1.3821(15)
C^{41}	C^{50}	1.4336(15)
C^{42}	C^{43}	1.4093(17)
C^{43}	C^{44}	1.3669(18)
C^{44}	C^{45}	1.4188(16)
C^{45}	C^{46}	1.4187(17)
C^{45}	C^{50}	1.4266(15)
C^{46}	C^{47}	1.3678(18)
C^{47}	C^{48}	1.4086(19)
C^{48}	C^{49}	1.3729(17)
C^{49}	C^{50}	1.4231(15)
C^{51}	C^{52}	1.3752(17)
C^{51}	C^{60}	1.4405(16)
C^{52}	C^{53}	1.4121(17)
C^{53}	C^{54}	1.370(2)
C^{54}	C^{55}	1.418(2)
C^{55}	C^{56}	1.4277(19)
C^{55}	C^{60}	1.4264(19)
C^{56}	C^{57}	1.369(3)
C^{57}	C^{58}	1.397(3)
C^{58}	C^{59}	1.3773(19)
C^{59}	C^{60}	1.4250(19)

----

[1]1-x,1-y,-z

Table S11: Bond Angles in ° for **2023NCS0627_1a**.

Atom	Atom	Atom	Angle/°
Er^{11}	Er^{1}	O^{11}	83.26(3)
Er^{11}	Er^{1}	O^{1}	82.16(3)
Er^{11}	Er^{1}	O^{11}	38.55(5)
Er^{11}	Er^{1}	O^{12}	91.51(8)
Er^{11}	Er^{1}	O^{21}	90.66(8)
Er^{11}	Er^{1}	O^{22}	37.65(5)
Er^{11}	Er^{1}	O^{31}	155.55(5)
Er^{11}	Er^{1}	O^{32}	151.11(5)
Er^{11}	Er^{1}	N^{10}	64.92(6)
Er^{11}	Er^{1}	N^{20}	63.99(6)
Er^{11}	Er^{1}	N^{30}	177.41(5)
O^{11}	Er^{1}	O^{1}	165.416(6)
O^{11}	Er^{1}	O^{11}	81.27(5)
O^{1}	Er^{1}	O^{11}	87.31(5)
O^{11}	Er^{1}	O^{12}	97.06(6)
O^{1}	Er^{1}	O^{12}	83.34(6)
O^{1}	Er^{1}	O^{21}	102.46(6)
O^{11}	Er^{1}	O^{21}	77.68(6)
O^{11}	Er^{1}	O^{22}	83.06(5)
O^{1}	Er^{1}	O^{22}	85.40(5)
O^{1}	Er^{1}	O^{31}	112.56(5)
O^{11}	Er^{1}	O^{31}	81.19(5)
O^{11}	Er^{1}	O^{32}	114.98(5)
O^{1}	Er^{1}	O^{32}	78.51(5)
O^{1}	Er^{1}	N^{10}	84.05(5)
O^{11}	Er^{1}	N^{10}	89.78(5)
O^{1}	Er^{1}	N^{20}	96.55(5)
O^{11}	Er^{1}	N^{20}	76.95(5)
O^{1}	Er^{1}	N^{30}	95.37(5)
O^{11}	Er^{1}	N^{30}	99.21(5)
O^{11}	Er^{1}	O^{12}	54.01(9)
O^{11}	Er^{1}	O^{21}	126.98(8)
O^{11}	Er^{1}	O^{22}	75.98(6)
O^{11}	Er^{1}	O^{31}	119.82(6)
O^{11}	Er^{1}	O^{32}	158.09(6)
O^{11}	Er^{1}	N^{10}	27.15(7)
O^{11}	Er^{1}	N^{20}	101.14(7)
O^{11}	Er^{1}	N^{30}	142.38(6)
O^{12}	Er^{1}	O^{21}	174.04(8)
O^{12}	Er^{1}	O^{32}	107.22(9)

Atom	Atom	Atom	Angle/°
O^{12}	Er^{1}	N^{10}	26.87(8)
O^{12}	Er^{1}	N^{20}	155.15(6)
O^{12}	Er^{1}	N^{30}	88.95(9)
O^{21}	Er^{1}	O^{32}	72.99(9)
O^{21}	Er^{1}	N^{10}	153.89(6)
O^{21}	Er^{1}	N^{20}	26.69(7)
O^{21}	Er^{1}	N^{30}	89.13(8)
O^{22}	Er^{1}	O^{12}	129.03(9)
O^{22}	Er^{1}	O^{21}	53.65(8)
O^{22}	Er^{1}	O^{32}	118.83(6)
O^{22}	Er^{1}	N^{10}	102.57(7)
O^{22}	Er^{1}	N^{20}	27.13(7)
O^{22}	Er^{1}	N^{30}	141.63(6)
O^{31}	Er^{1}	O^{12}	71.92(9)
O^{31}	Er^{1}	O^{21}	104.23(8)
O^{31}	Er^{1}	O^{22}	155.36(6)
O^{31}	Er^{1}	O^{32}	53.33(6)
O^{31}	Er^{1}	N^{10}	96.21(7)
O^{31}	Er^{1}	N^{20}	129.47(7)
O^{31}	Er^{1}	N^{30}	26.64(6)
O^{32}	Er^{1}	N^{10}	133.03(7)
O^{32}	Er^{1}	N^{20}	97.04(7)
O^{32}	Er^{1}	N^{30}	26.71(6)
N^{10}	Er^{1}	N^{20}	128.28(7)
N^{10}	Er^{1}	N^{30}	115.69(7)
N^{20}	Er^{1}	N^{30}	115.72(7)
O^{1}	P^{1}	C^{1}	110.86(5)
O^{1}	P^{1}	C^{11}	110.98(5)
O^{1}	P^{1}	C^{21}	111.69(5)
C^{11}	P^{1}	C^{1}	107.46(5)
C^{21}	P^{1}	C^{1}	107.27(5)
C^{21}	P^{1}	C^{11}	108.40(5)
Er^{11}	O^{1}	Er^{1}	14.586(6)
P^{1}	O^{1}	Er^{1}	173.18(5)
P^{1}	O^{1}	Er^{11}	172.22(5)
N^{10}	O^{11}	Er^{1}	95.11(15)
N^{10}	O^{12}	Er^{1}	95.18(17)
N^{20}	O^{21}	Er^{1}	94.59(17)
N^{20}	O^{22}	Er^{1}	95.18(15)
N^{30}	O^{31}	Er^{1}	96.47(13)
N^{30}	O^{32}	Er^{1}	94.84(13)
O^{11}	N^{10}	Er^{1}	57.74(11)
O^{12}	N^{10}	Er^{1}	57.95(13)
O^{12}	N^{10}	O^{11}	115.6(2)
O^{13}	N^{10}	Er^{1}	177.33(17)
O^{13}	N^{10}	O^{11}	122.3(3)
O^{13}	N^{10}	O^{12}	122.1(2)
O^{21}	N^{20}	Er^{1}	58.72(14)
O^{21}	N^{20}	O^{22}	115.9(2)
O^{22}	N^{20}	Er^{1}	57.69(12)
O^{23}	N^{20}	Er^{1}	171.91(19)
O^{23}	N^{20}	O^{21}	122.6(2)
O^{23}	N^{20}	O^{22}	121.6(3)
O^{31}	N^{30}	Er^{1}	56.89(10)
O^{32}	N^{30}	Er^{1}	58.44(10)
O^{32}	N^{30}	O^{31}	115.27(18)
O^{33}	N^{30}	Er^{1}	177.35(16)
O^{33}	N^{30}	O^{31}	123.2(2)
O^{33}	N^{30}	O^{32}	121.5(2)
C^{2}	C^{1}	P^{1}	117.84(8)
C^{2}	C^{1}	C^{10}	120.03(9)

Atom	Atom	Atom	Angle/°
C^{10}	C^{1}	P^{1}	122.13(8)
C^{1}	C^{2}	C^{3}	121.17(11)
C^{4}	C^{3}	C^{2}	119.74(11)
C^{3}	C^{4}	C^{5}	121.10(10)
C^{4}	C^{5}	C^{6}	120.98(11)
C^{4}	C^{5}	C^{10}	119.80(10)
C^{6}	C^{5}	C^{10}	119.13(12)
C^{7}	C^{6}	C^{5}	120.91(11)
C^{6}	C^{7}	C^{8}	120.23(11)
C^{9}	C^{8}	C^{7}	120.49(12)
C^{8}	C^{9}	C^{10}	120.79(11)
C^{5}	C^{10}	C^{1}	117.82(10)
C^{9}	C^{10}	C^{1}	123.76(10)
C^{9}	C^{10}	C^{5}	118.38(10)
C^{12}	C^{11}	P^{1}	118.71(8)
C^{12}	C^{11}	C^{20}	120.18(10)
C^{20}	C^{11}	P^{1}	121.09(8)
C^{11}	C^{12}	C^{13}	120.84(11)
C^{14}	C^{13}	C^{12}	120.19(11)
C^{13}	C^{14}	C^{15}	120.92(11)
C^{14}	C^{15}	C^{16}	120.71(11)
C^{14}	C^{15}	C^{20}	119.84(11)
C^{16}	C^{15}	C^{20}	119.45(11)
C^{17}	C^{16}	C^{15}	121.00(12)
C^{16}	C^{17}	C^{18}	119.85(12)
C^{19}	C^{18}	C^{17}	120.85(12)
C^{18}	C^{19}	C^{20}	120.78(11)
C^{15}	C^{20}	C^{11}	118.01(10)
C^{19}	C^{20}	C^{11}	123.94(10)
C^{19}	C^{20}	C^{15}	118.05(10)
C^{22}	C^{21}	P^{1}	118.12(8)
C^{22}	C^{21}	C^{30}	119.92(10)
C^{30}	C^{21}	P^{1}	121.96(8)
C^{21}	C^{22}	C^{23}	121.28(11)
C^{24}	C^{23}	C^{22}	119.82(11)
C^{23}	C^{24}	C^{25}	120.94(11)
C^{24}	C^{25}	C^{26}	120.64(12)
C^{24}	C^{25}	C^{30}	120.03(10)
C^{26}	C^{25}	C^{30}	119.31(12)
C^{27}	C^{26}	C^{25}	120.68(13)
C^{26}	C^{27}	C^{28}	120.40(12)
C^{29}	C^{28}	C^{27}	120.53(13)
C^{28}	C^{29}	C^{30}	120.49(12)
C^{25}	C^{30}	C^{21}	117.96(10)
C^{29}	C^{30}	C^{21}	123.46(10)
C^{29}	C^{30}	C^{25}	118.55(10)
O^{2}	P^{2}	C^{31}	111.72(5)
O^{2}	P^{2}	C^{41}	115.54(5)
O^{2}	P^{2}	C^{51}	113.01(5)
C^{41}	P^{2}	C^{31}	104.47(5)
C^{51}	P^{2}	C^{31}	107.66(5)
C^{51}	P^{2}	C^{41}	103.65(5)
C^{32}	C^{31}	P^{2}	120.55(9)
C^{32}	C^{31}	C^{40}	119.38(10)
C^{40}	C^{31}	P^{2}	119.79(8)
C^{31}	C^{32}	C^{33}	121.42(11)
C^{34}	C^{33}	C^{32}	120.04(12)
C^{33}	C^{34}	C^{35}	120.78(11)
C^{34}	C^{35}	C^{36}	121.12(11)
C^{34}	C^{35}	C^{40}	119.59(11)
C^{36}	C^{35}	C^{40}	119.27(12)

Atom	Atom	Atom	Angle/°
C^{37}	C^{36}	C^{35}	120.87(12)
C^{36}	C^{37}	C^{38}	120.00(12)
C^{39}	C^{38}	C^{37}	120.56(13)
C^{38}	C^{39}	C^{40}	120.98(11)
C^{35}	C^{40}	C^{31}	118.75(11)
C^{39}	C^{40}	C^{31}	122.92(10)
C^{39}	C^{40}	C^{35}	118.31(11)
C^{42}	C^{41}	P^{2}	117.96(9)
C^{42}	C^{41}	C^{50}	119.33(10)
C^{50}	C^{41}	P^{2}	122.63(8)
C^{41}	C^{42}	C^{43}	121.83(11)
C^{44}	C^{43}	C^{42}	119.83(11)
C^{43}	C^{44}	C^{45}	120.57(11)
C^{44}	C^{45}	C^{50}	120.00(11)
C^{46}	C^{45}	C^{44}	120.34(11)
C^{46}	C^{45}	C^{50}	119.65(11)
C^{47}	C^{46}	C^{45}	120.84(12)
C^{46}	C^{47}	C^{48}	119.63(12)
C^{49}	C^{48}	C^{47}	121.30(11)
C^{48}	C^{49}	C^{50}	120.49(11)
C^{45}	C^{50}	C^{41}	118.40(10)
C^{49}	C^{50}	C^{41}	123.54(10)
C^{49}	C^{50}	C^{45}	118.06(10)
C^{52}	C^{51}	P^{2}	119.97(9)
C^{52}	C^{51}	C^{60}	120.07(11)
C^{60}	C^{51}	P^{2}	119.96(9)
C^{51}	C^{52}	C^{53}	121.30(12)
C^{54}	C^{53}	C^{52}	119.84(13)
C^{53}	C^{54}	C^{55}	120.96(12)
C^{54}	C^{55}	C^{56}	121.35(13)
C^{54}	C^{55}	C^{60}	119.72(12)
C^{60}	C^{55}	C^{56}	118.92(14)
C^{57}	C^{56}	C^{55}	120.87(15)
C^{56}	C^{57}	C^{58}	120.24(13)
C^{59}	C^{58}	C^{57}	120.96(16)
C^{58}	C^{59}	C^{60}	120.56(15)
C^{55}	C^{60}	C^{51}	118.10(12)
C^{59}	C^{60}	C^{51}	123.46(12)
C^{59}	C^{60}	C^{55}	118.43(12)

––––

[1]1-x,1-y,-z

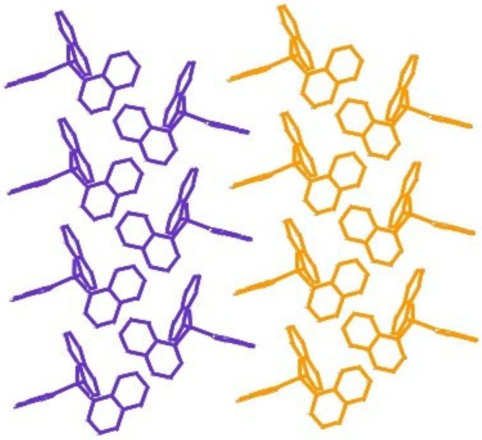

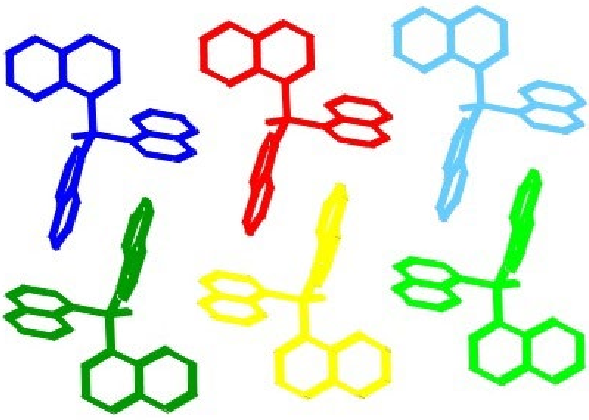

Sheet edge – face

Sheet edge - face

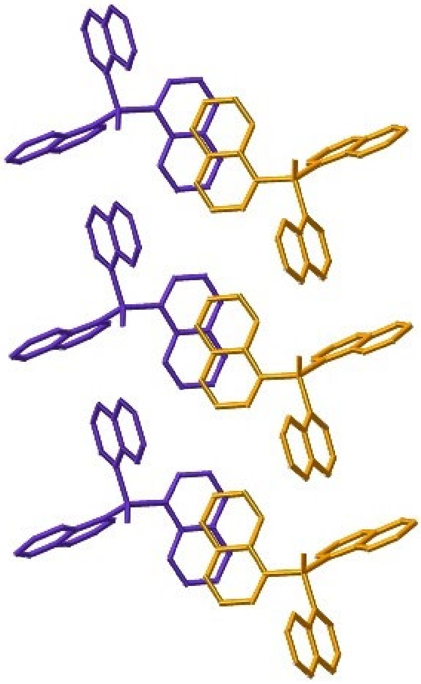

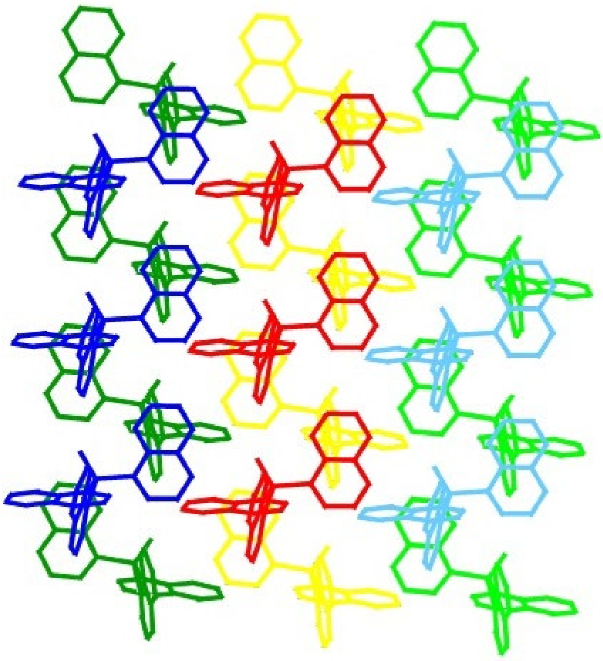

Face - face

Sheet edge - face

Figure S6 Packing diagrams for Nap_3PO
